# The optimal timing and intervention to reduce mortality for necrotizing pancreatitis: a systematic review and network meta-analysis

**DOI:** 10.1186/s13017-023-00479-7

**Published:** 2023-01-27

**Authors:** Yang Yang, Yu Zhang, Shuaiyong Wen, Yunfeng Cui

**Affiliations:** 1 Department of Surgery, Tianjin Medical University, Tianjin, 300070 China; 2 Department of Surgery, Tianjin Nankai Hospital, Nankai Clinical School of Medicine, 122 Sanwei Road, Nankai District, Tianjin, 300110 China

**Keywords:** Necrotizing pancreatitis, Network meta-analysis, Intervention, Randomized controlled trials, Mortality

## Abstract

**Background:**

A series of randomized controlled trials have investigated the efficacy and safety of different timings of interventions and methods of intervention. However, the optimal treatment strategy is not yet clear.

**Methods:**

We searched PubMed, EMBASE, ClinicalTrials.gov and the Cochrane Library until November 30, 2022. A systematic review and Bayesian network meta-analysis were performed following the Preferred Reporting Items for Systematic Reviews and Meta-Analyses (PRISMA) guidelines. Trials comparing different treatment strategies for necrotizing pancreatitis were included. This study was registered in the Prospective Register of Systematic Reviews (CRD42022364409) to ensure transparency.

**Results:**

We analyzed a total of 10 studies involving 570 patients and 8 treatment strategies. Although no statistically significant differences were identified comparing odds ratios, trends were confirmed by the surface under the cumulative ranking (SUCRA) scores. The interventions with a low rate of mortality were delayed surgery (DS), delayed surgical step-up approach (DSU) and delayed endoscopic step-up approach (DEU), while the interventions with a low rate of major complications were DSU, DEU and DS. According to the clustered ranking plot, DSU performed the best overall in reducing mortality and major complications, while DD performed the worst. Analysis of the secondary endpoints confirmed the superiority of DEU and DSU in terms of individual components of major complications (organ failure, pancreatic fistula, bleeding, and visceral organ or enterocutaneous fistula), exocrine insufficiency, endocrine insufficiency and length of stay. Overall, DSU was superior to other interventions.

**Conclusion:**

DSU was the optimal treatment strategy for necrotizing pancreatitis. Drainage alone should be avoided in clinical practice. Any interventions should be postponed for at least 4 weeks if possible. The step-up approach was preferred.

**Supplementary Information:**

The online version contains supplementary material available at 10.1186/s13017-023-00479-7.

## Introduction

Acute pancreatitis (AP) is an inflammatory disorder of the pancreas with 10% to 40% mortality [1, 2]. There are 2 major forms of acute pancreatitis: interstitial and necrotizing [3]. Approximately 20% of AP patients progress to necrotizing pancreatitis (NP), which is associated with acute necrotic collections (ANC) or walled-off necrosis (WON), with a significant mortality risk as high as 20–30% [4–6]. In patients developing an NP, intervention is required in 40–63% of patients [7, 8].

Over the last decade, the management of NP patients has evolved dramatically. Traditional surgical debridement promotes the removal of infected and necrotic tissue and is considered a maximally invasive procedure with a high incidence of complications and high mortality [9, 10]. As an alternative to surgery, minimally invasive endoscopic transluminal interventions have emerged [11, 12]. It is characterized by a shorter length of hospital stay, fewer complications and lower mortality rates [11]. The current standard treatment for necrotizing pancreatitis has gradually shifted toward a step-up approach, which starts with infected or necrotic tissue drainage, followed by minimally invasive interventions, including laparoscopic cystogastrostomy and video-assisted retroperitoneal debridement (VARD) [13, 14]. Finally, endoscopic transluminal necrosectomy or surgical necrosectomy will be performed if required [15]. In addition, transluminal drainage, including percutaneous drainage and endoscopic ultrasound (EUS)-guided drainage, is clinically effective [16].

International evidence-based guidelines recommend primary conservative therapy and postponement of invasive interventions until necrosis becomes walled off and liquified, which usually requires 3–4 weeks [8, 17, 18]. However, evidence on the effectiveness of delayed interventions from the open surgery era is limited and inconsistent [16, 19]. In 2016, an international expert survey reported that 45% advised early interventions as soon as necrotizing pancreatitis was diagnosed [20]. With the advent of minimally invasive techniques, it is argued that patients could benefit more from postponement [21].

There is still no consensus on which is the superior treatment strategy for necrotizing pancreatitis. Although several conventional meta-analyses that compared endoscopic interventions versus minimally invasive surgery, retroperitoneal versus open intraperitoneal necrosectomy, endoscopic versus surgical intervention and early versus delayed minimally invasive approaches have been published previously and found benefits of the endoscopic approach and delayed interventions, they were limited by the availability of pairwise comparisons between interventions and provided no comprehensive results [15, 22–25]. Empirical studies have suggested that network meta-analyses (NMA) can yield more comprehensive and precise comparisons of available interventions for necrotizing pancreatitis beyond conventional meta-analyses [26]. Previous network meta-analyses suggested that a step-up approach with endoscopic debridement was the first choice for suspected infected pancreatic necrosis; however, they combined different interventions into a single category, which contributed to inherent heterogeneity [27].

In this study, we conducted a comprehensive Bayesian network meta-analysis (NMA) of randomized controlled trials (RCTs) to investigate the optimal timing and intervention for necrotizing pancreatitis comparing safety and efficacy through direct and indirect evidence.

## Methods

This review followed the Cochrane Handbook of Systematic Reviews and Interventions and the Preferred Reporting Items for Systematic Reviews and Meta-Analyses (PRISMA) statement reporting systematic reviews and network meta-analyses [28, 29]. This review was registered prospectively in PROSPERO (CRD42022364409).

### Eligibility criteria

Only trials fulfilling the following The Population, Intervention, Comparator, Outcome and Study design (PICOs) criteria were eligible to be included. (1) Patients: Patients with confirmed or suspected necrotizing pancreatitis were eligible. (2) Intervention and control: All studies consisted of two or more intervention arms for necrotizing pancreatitis, which were clustered based on timing (early, within 72 h, or delayed, after 4 weeks), approach (drainage, endoscopic or surgical) and application of the step-up strategy (yes or no). An approach with drainage to delay debridement as long as possible was considered a step-up strategy. The intervention arms included early drainage (ED), delayed drainage (DD), early surgery (ES), early endoscopic step-up approach (EEU), delayed endoscopic step-up approach (DEU), delayed surgical step-up approach (DSU), delayed endoscopic debridement (DE), delayed surgery (DS), early surgical step-up approach (ESU), and early endoscopic debridement (EE). 3) Outcomes: We only included articles that reported at least one of the primary endpoints (mortality or a composite of major complications). 4) Study design: Only randomized controlled trials (RCTs) were included.

### Information sources and search strategy

Two of the authors (YY and ZY) searched PubMed, Embase, Cochrane Library and ClinicalTrials.gov independently. All potentially relevant studies were identified using a combination of MeSH terms, Emtree terms, and keywords that describe “necrotizing pancreatitis.” Detailed information about the search strategies in each database is supplied in Additional file 1: Appendix S1. To identify additional relevant studies, we reviewed the references in the retrieved articles. The search had no language restrictions and included the period from the inception of each database to November 30, 2022. All retrieved literature works were collected in the EndNote database (Version X9).

### Selection process

Two of the authors (YY and WSY) screened the studies for eligibility independently. We excluded abstracts only, review summaries, editorials, case reports, protocols, uncontrolled studies and non-randomized controlled trials. For studies published in languages other than English, we obtained the translation via Google Translate to determine potential eligibility. Discrepancies between the two investigators were resolved with a third reviewer (ZY).

### Data collection process

Two of the authors (YY and WSY) independently extracted data from the included studies using a predefined form in Excel 2019 (Microsoft, Redmond, WA, USA) and then compared them. Discrepancies between the two investigators were resolved with a third reviewer (ZY).

### Data items

The extracted data were as follows:

1. General information: the first author’s name, year of publication, country, study period and follow-up time;
2. Baseline data: number of patients; age; gender; etiology; extent of necrosis; CT severity index; and APACHE II (Acute Physiology and Chronic Health Evaluation II);
3. Intervention arms: ED, DD, ES, EEU, DEU, DSU, DE and DS;
4. Primary and secondary endpoints: The primary endpoints were mortality and a composite of major complications. Major complications consisted of organ failure, pancreatic fistula, bleeding, visceral organ or enterocutaneous fistula, etc. Multiple events in the same patient were considered one endpoint. All the data on the composite of major complications used in the analysis were mentioned in the original article. The secondary endpoints were individual components of major complications (organ failure, pancreatic fistula, bleeding, and visceral organ or enterocutaneous fistula), length of hospital stay, exocrine insufficiency and endocrine insufficiency. The definitions of endpoints are presented in Additional file 1: Appendix S2.

### Study risk-of-bias assessment

Risk of bias was assessed using the Cochrane risk-of-bias tool 2.0 version in terms of random sequence generation (selection bias), allocation concealment (selection bias), blinding of participants and personnel (performance bias), blinding of outcome assessment (detection bias), incomplete outcome data (attrition bias), selective reporting (reporting bias) and other bias. The study quality assessment was categorized into low risk, unclear risk and high risk. Two of the authors (YY and ZY) assessed all studies independently. Discrepancies between the two investigators were resolved with a third reviewer (WSY).

### Effect measures and synthesis methods

Endpoints (outcome measures) were presented as the number and proportion for dichotomous data and means ± standard deviations (SD) for continuous data. If data were presented in the original paper other than mean and SD, we recalculated these data using the methods described by Hozo et al. and Higgins and Green [29, 30].

Traditional pairwise meta-analysis was performed to provide direct evidence using the statistical software Review Manager 5.3. We chose random effects models that assumed that the true effects were not identical in different studies. An inverse variance method was used for continuous data and a Mantel–Haenszel method for dichotomous data. The results were presented as odds ratios (ORs) with 95% confidence intervals (CIs) for dichotomous data and mean difference (MD) with 95% CIs for continuous data.

To incorporate indirect evidence into the assessment, a Bayesian network meta-analysis (NMA) was conducted using the statistical software R (version 4.2.1) with GeMTC package (version 1.0–1) and JAGS (version 4.3.0). A network plot was used to visualize all direct and indirect comparisons. The solid line represented direct comparisons, while the dotted line represented indirect comparisons. The size of the node represented the sample size for each intervention arm, and the width of the lines was proportional to the number of trials comparing the connected intervention arms. We used the random effects model to calculate all comparisons in the network and at least 20,000 simulation iterations and a burn-in of 5000 iterations were repeated until convergence was reached. The models were optimized and estimates were obtained using Markov chain Monte Carlo methods. This process was intended to obtain a posterior distribution, with model convergence of the iterations being evaluated and visualized using trace plots and Brooks–Gelman–Rubin diagnostic. OR corresponding 95% credible intervals (CrI) were obtained through posterior distribution.

The Bayesian approach also provided overall ranking probabilities, making it possible to rank each outcome measurement from the best to the worst, and was then visualized by calculating the surface under the cumulative ranking curves (SUCRA) on the basis of the ranking profiles.

Network meta-analysis relies on the assumption of transitivity to make indirect comparisons [31]. We ensured that the inclusion criteria of individual study were similar and assumed that all participants in the network meta-analysis could be randomly allocated to any of the interventions. This was a reasonable assumption considering that all studies included within this network meta-analysis were RCTs. To assess transitivity, we explored the distribution of study characteristics among treatment comparisons across included studies with a meta-regression analysis using STATA software (e.g., gender ratio, mean age, country, publication year, presence of organ failure and extent of necrosis ≥ 30%) to determine whether the results were affected by study characteristics [32].

Consistency, whereby the treatment effect estimated from direct comparisons was consistent with that estimated from indirect comparisons, was assessed using the node-splitting method by checking if *p* < 0.05 [33–35].

We assumed that the different comparisons in the network meta-analysis shared the same heterogeneity. Heterogeneity was assessed using the Cochrane Q test (pairwise meta-analysis) and *I*^2^ statistics (network meta-analysis) [33, 36]. *I*^2^ > 50% indicated the existence of heterogeneity [36].

### Reporting bias and certainty assessment

Egger’s test was used for publication bias (across-study) when ≥ 10 studies were included, while a comparison-adjusted funnel plot with accompanying Egger’s test for asymmetry was used for publication bias (across-study). *p* value of less than 0.10 was considered to indicate significant asymmetry and publication bias [29, 37]. Publication bias could not be evaluated in other endpoints because the number of included studies was less than 10 [29].

The certainty of evidence was assessed using the Grading of Recommendations Assessment, Development, and Evaluation (GRADE) approach with the recommendation of the Confidence in Network Meta-analysis (CINeMA) [38]. The assessment included within-study bias, reporting bias, indirectness, imprecision, heterogeneity, and incoherence. The final confidence rating consisted of four main descriptors: high, moderate, low and very low. Two of the authors (YY and WSY) assessed all studies independently. Discrepancies between the two investigators were resolved with a third reviewer (ZY).

## Results

### Study selection

**Fig. 1:**
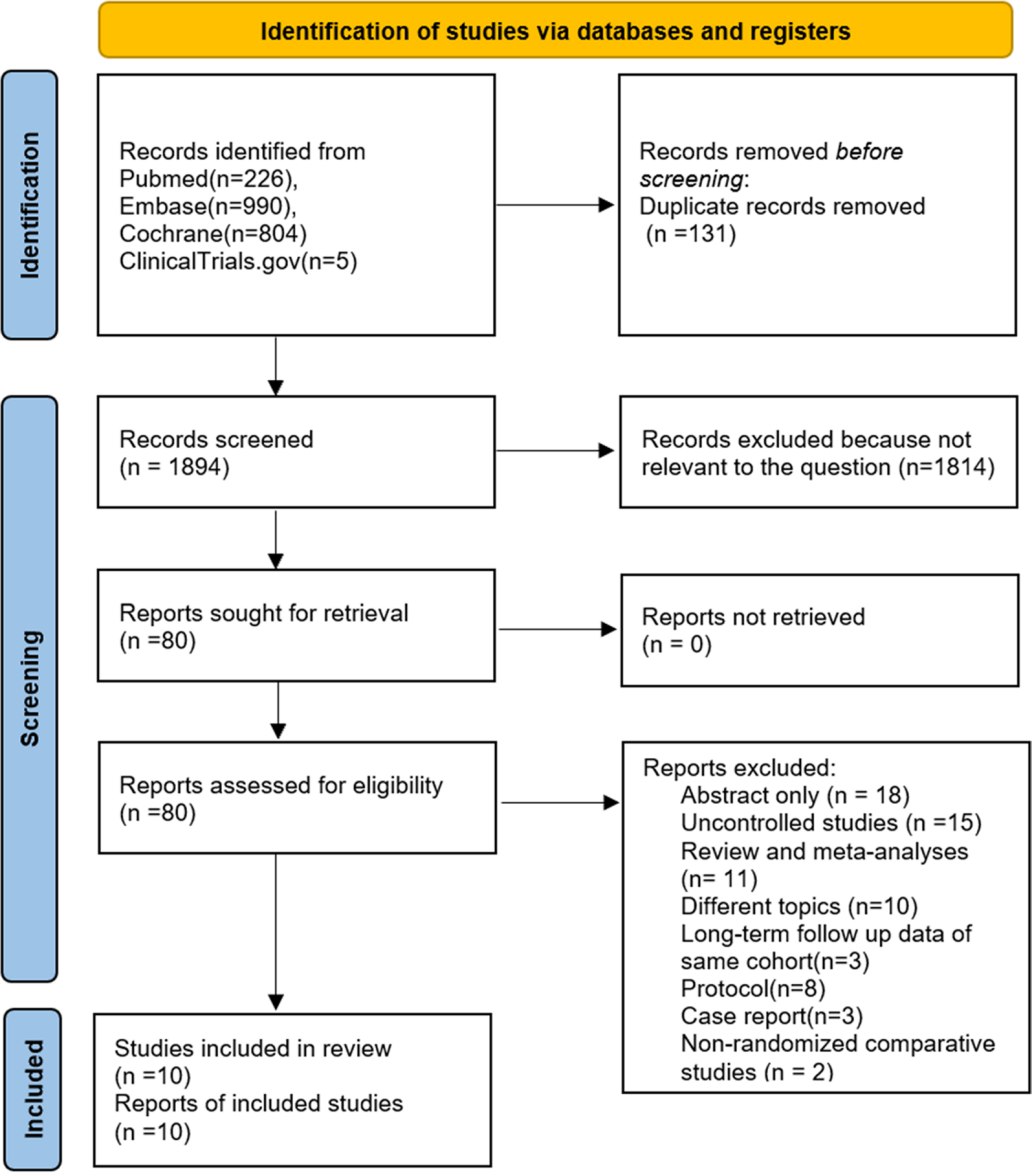
PRISMA flowchart

A total of 2025 articles were identified in accordance with the search strategy, and 131 duplicate citations were removed before screening. Of the remaining records, 1814 were excluded after title and abstract screening because of irrelevance. Seventy references were excluded after full-text screening. The reasons were as follows: abstract only (14 studies), uncontrolled studies (15 studies), traditional pairwise review and meta-analyses (11 studies), different topics did not meet the inclusion criteria because they were not clustered based on timing, approach and application of the step-up strategy (10 studies), protocol (8 studies), case reports (3 studies), studies reporting long-term follow-up outcomes of included studies (3 studies) and non-RCTs (2 studies). We did not identify any non-English articles that met our inclusion criteria. Finally, 10 RCTs were eligible for quality assessment and quantitative synthesis [39–48]. A PRISMA flowchart is presented in Fig. 1.

### Study characteristics

The 10 included studies were published from 1984 to 2021, with their respective studies starting from 1982 to 2019 across 6 countries [39–48] (Table 1). Ten studies [39–48] were designed as two-arm randomized controlled trials. A total of 570 participants with necrotizing pancreatitis were randomly assigned to one of the 8 interventions: 4 studies [39, 40, 47, 48] for early drainage (ED), 2 studies [47, 48] for delayed drainage (DD), 5 studies [39–42, 46] for early surgery (ES), 1 study [46] for early endoscopic step-up approach (EEU), 1 study [45] for delayed endoscopic step-up approach (DEU), 3 studies [42, 43, 45] for delayed surgical step-up approach (DSU), 1 study [44] for delayed endoscopic debridement (DE) and 3 studies [41, 43, 44] for delayed surgery (DS). The proportion of males in these studies ranged from 58 to 90.9%, and the mean age of patients ranged from 35.7 to 63 years. The etiology of necrotizing pancreatitis was reported in 7 studies [41, 43–48], and biliary pancreatitis was the most commonly identified etiology (53.2%, 235/442). The detailed baseline characteristics are shown in Additional file 1: Appendix S3.

**Table 1:** Characteristics of included trials within the network meta-analysis

Author, year	Country	Study period	Study design	Follow-up	Comparison	Size	Age, mean ± SD	Gender, male, n (%)
Ke, L., 2021 [48]	China	2018–2019	Prospective, single-center, randomized controlled trial	90 Days	ED	15	38 ± 18	11 (73.3%)
					DD	15	40 ± 16	9 (60%)
Boxhoorn, L.,2021 [47]	Netherlands	2015–2019	Prospective, multicenter, randomized, controlled superiority trial	3 Months/6 months	ED	55	60 ± 14	32 (58%)
					DD	49	59 ± 11	32 (65%)
Bang, J., 2019 [46]	The USA	2014–2017	Prospective, single-center, randomized trial	6 Weeks/6 months	ES	32	52.9 ± 14.2	21 (65.6%)
					EEU	34	55.6 ± 14.2	22 (64.7%)
van Brunschot, 2018 [45]	Netherlands	2011–2015	Prospective, multicenter randomized, superiority trial	3 Months/6 months	DEU	51	63 ± 14	34 (67%)
					DSU	47	60 ± 11	29 (62%)
Bakker, 2012 [44]	Netherlands	2008–2010	Prospective, randomized controlled assessor-blinded clinical trial	3 Months/6 months	DE	10	60 ± 22	8 (80%)
					DS	10	60 ± 19	6 (60%)
van Santvoort, 2010 [43]	Netherlands	2005–2008	Prospective, multicenter randomized controlled superiority trial	3 Months/6 months	DS	45	57.4 ± 2.0	33 (73%)
					DSU	43	57.6 ± 2.1	31 (72%)
Litvin A, 2010 [42]	Belarus	2004–2008	Prospective, multicenter randomized trial	NR	DSU	37	NR	NR
					ES	35	NR	NR
Mier, J., 1997 [41]	Mexico	1990–1993	Prospective, single-center, randomized trial	NR	ES	25	42 ± 16	15 (60%)
					DS	11	42 ± 12	7 (63.6%)
Schröder, T, 1991 [40]	Finland	1984–1988	Prospective, single-center, randomized trial	NR	ES	11	40.5 ± 7.5	10 (90.9%)
					ED	10	35.7 ± 6.8	9 (90%)
Kivilaakso, E, 1984 [39]	Finland	1997–1982	Prospective, single-center, randomized trial	NR	ES	18	38.4 ± 10.2	16 (88.9%)
					ED	17	39.7 ± 10	14 (82.4%)

*DD* delayed drainage, *DE* delayed endoscopic debridement, *DEU* delayed endoscopic step-up approach, *DS* delayed surgery, *DSU* delayed surgical step-up approach, *ED* early drainage, *EEU* early endoscopic step-up approach, *ES* early surgery, *NR* not state, *SD* standard deviation

Ten studies [39–48] reported morality. Eight studies [39–44, 46, 47] reported major complications. Nine studies [39, 40, 42–48] reported the length of hospital stay. Seven studies [42–48] compared bleeding, and six studies [43–48] reported organ failure, pancreatic fistula, visceral organ or enterocutaneous fistula, exocrine insufficiency, and endocrine insufficiency. (Additional file 1: Appendix S4).

### Risk of bias in studies

**Fig. 2:**
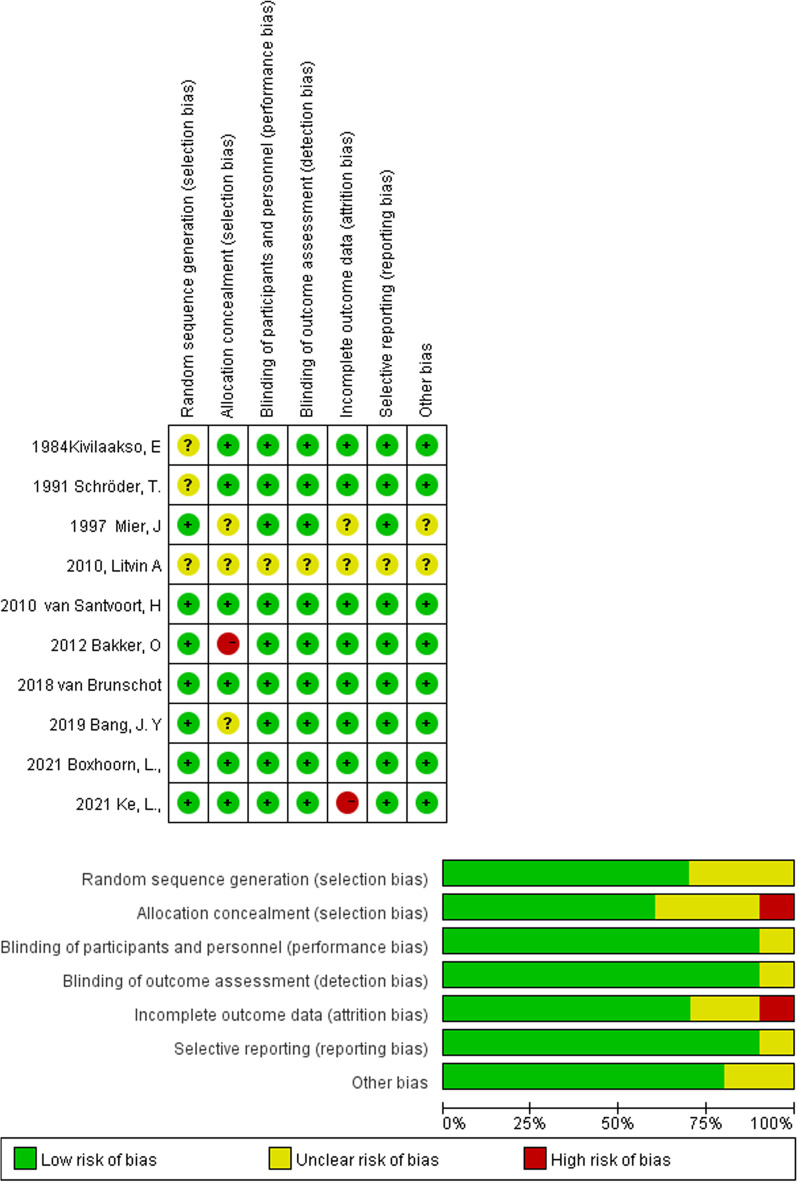
Assessment of risk of bias in the trials used in analysis using the Cochrane Collaborations tool

The quality assessment of individual studies is presented in Fig. 2. Using the Cochrane Collaboration tool, the overall risk of bias was low to moderate. Seven (70%) studies [41, 43–48] clearly described the generation of random sequences, and 6 (60%) studies [39, 40, 43, 45, 47, 48] had adequate allocation concealment procedures. The primary and secondary endpoints were rarely affected by the binding of participants and personnel. In addition, one study [48] had incomplete outcome data.

### Primary endpoint

**Fig. 3:**
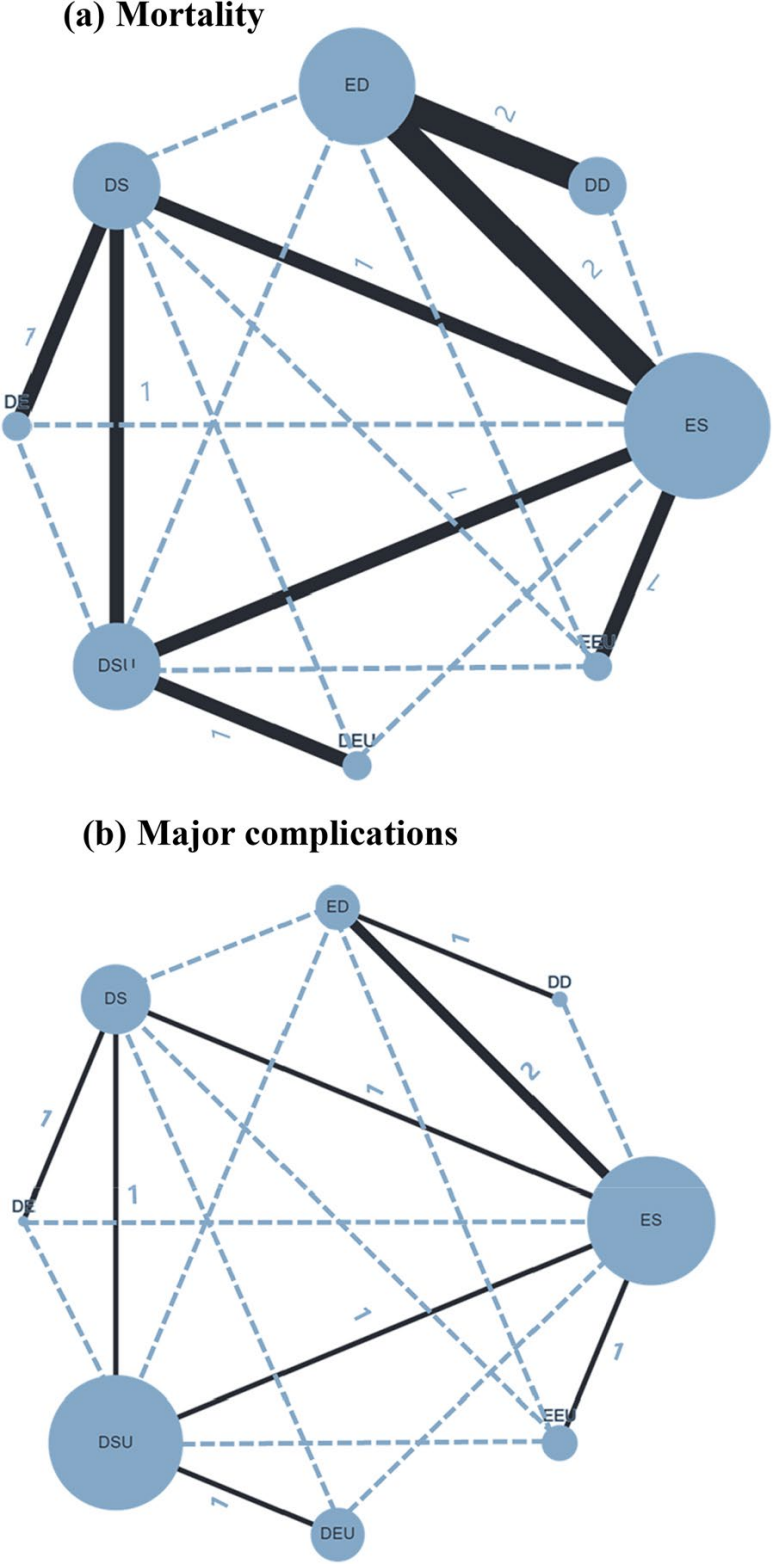
Network of included trials comparing interventions for necrotizing pancreatitis. **a** Mortality. **b** Major complications. DD, delayed drainage; DE, delayed endoscopic debridement; DEU, delayed endoscopic step-up approach; DS, delayed surgery; DSU, delayed surgical step-up approach; ED, early drainage; EEU, early endoscopic step-up approach; ES, early surgery

**Fig. 4:**
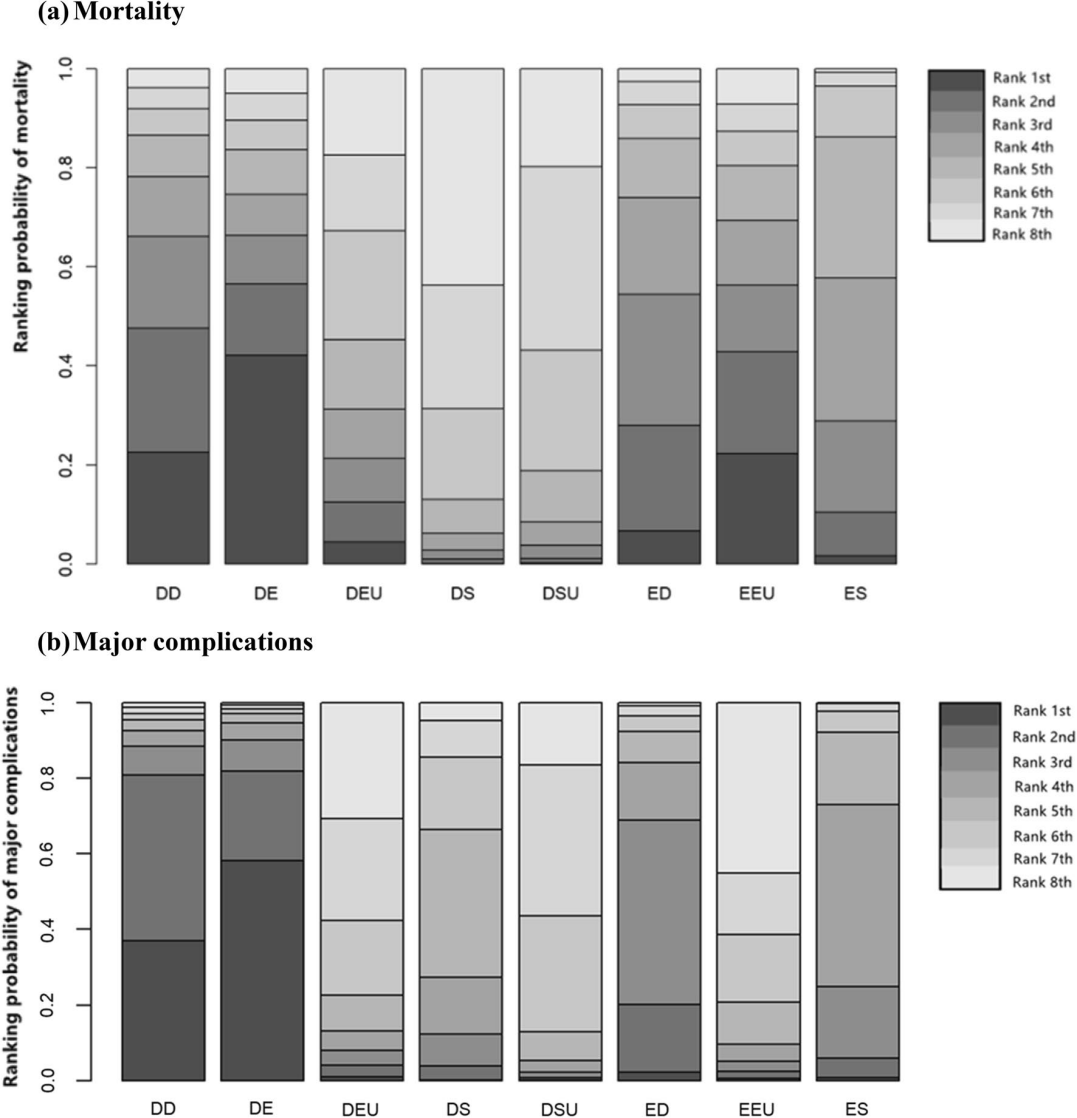
Ranking probability of mortality and major complications for different interventions for necrotizing pancreatitis. **a** Mortality. **b** Major complications. DD, delayed drainage; DE, delayed endoscopic debridement; DEU, delayed endoscopic step-up approach; DS, delayed surgery; DSU, delayed surgical step-up approach; ED, early drainage; EEU, early endoscopic step-up approach; ES, early surgery

The mortality of necrotizing pancreatitis participants was reported in all 10 studies [39–48]. Figure 3a displays the network of all the interventions included in this network meta-analysis, the number of RCTs comparing different interventions and the sample size of each intervention. Table 2a presents the direct and indirect evidence using both traditional pairwise meta-analysis and Bayesian network meta-analysis. Accordingly, patients with ED and DD were more likely to obtain greater survival benefits than those who received ES from pairwise meta-analysis, (OR 2.56, 95% CI 2.06–25.03; OR 3.13, 95% CI 2.19–14.30, respectively) [49]. In addition, ES was discerned to offer a marked higher risk of mortality in comparison with DSU and DS (OR 2.56, 95% CI 1.05–6.69; OR 3.39, 95% CI 1.30–15.96, respectively). The remaining direct comparisons were statistically insignificant. However, based on the SUCRA scores, DE had the highest probability of mortality (73%), followed by DD (70%), EEU (65%), ED (63%), ES (54%), DEU (37%), DSU (24%) and DS (16%). In other words, the top three interventions ranked by safety were DS, DSU and DEU (Fig. 4a).

**Table 2:**
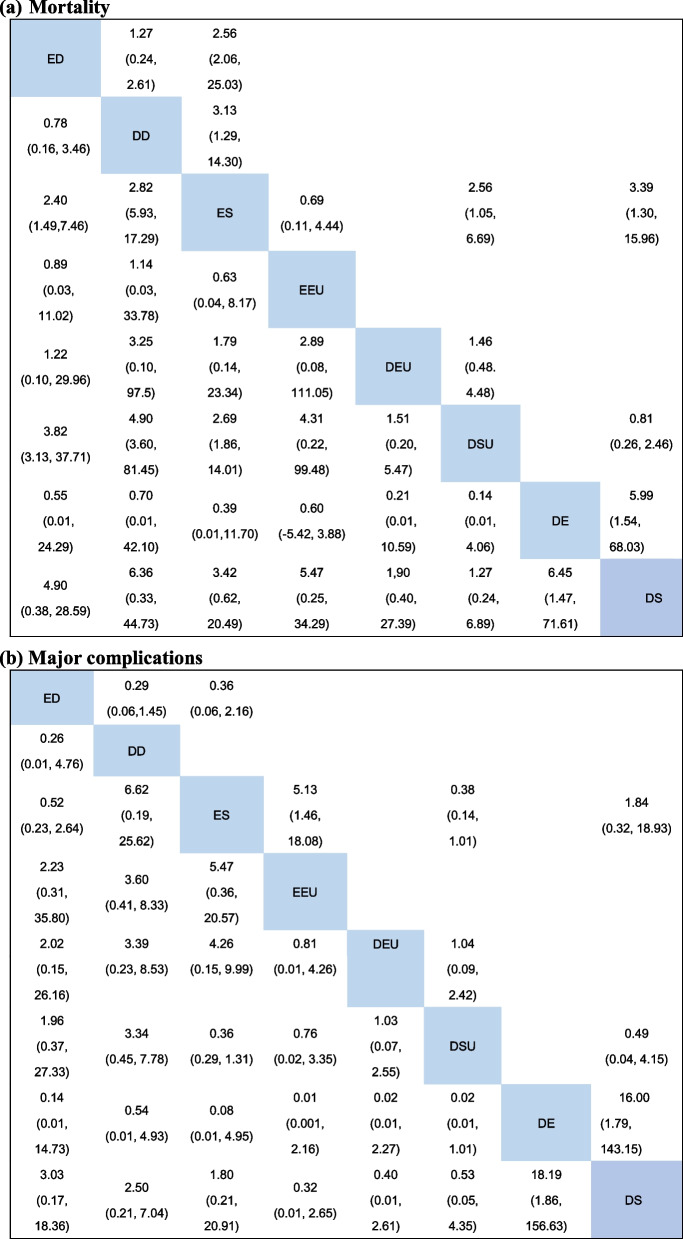
Direct and indirect evidence of primary endpoints in the network meta-analysis

ORs with 95% CrI below the diagonal were from the network meta-analysis, combining direct and indirect evidence, whereas ORs with 95% CI above the diagonal were from pairwise meta-analysis. ORs compared the column-defining and row-defining agents. Values > 1 indicated that the intervention for necrotizing pancreatitis in the corresponding column had a higher risk of mortality or major complications than those in the corresponding rows and values < 1 indicated a lower risk

*CIs* confidence intervals, *CrI* credible interval, *DD* delayed drainage, *DE* delayed endoscopic debridement, *DEU* delayed endoscopic step-up approach, *DS* delayed surgery, *DSU* delayed surgical step-up approach, *ED* early drainage, *EEU* early endoscopic step-up approach, *ES* early surgery, *ORs* odds ratios

A total of 9 studies [39, 40, 42–48] reported major complications (Fig. 3b). Multiple events in the same patient were considered one endpoint. All the data on the composite of major complications used in the analysis were mentioned in the original article. As given in Table 2b, major complications were more common in the ES group than in the EEU group (OR 5.13, 95% CI 1.46–18.08). For network meta-analysis, we found that DD was most likely to rank first for major complications rates (85%), followed by ED (77%), DE (66%), ES (56%), EEU (38%), DS (33%), DEU (24%) and DSU (21%), using SUCRA (Fig. 4b). Regarding safety, DSU, DEU and DS ranked among the top three interventions for necrotizing pancreatitis. However, compared to ES, there were no differences in the major complication rates in other interventions.

**Fig. 5:**
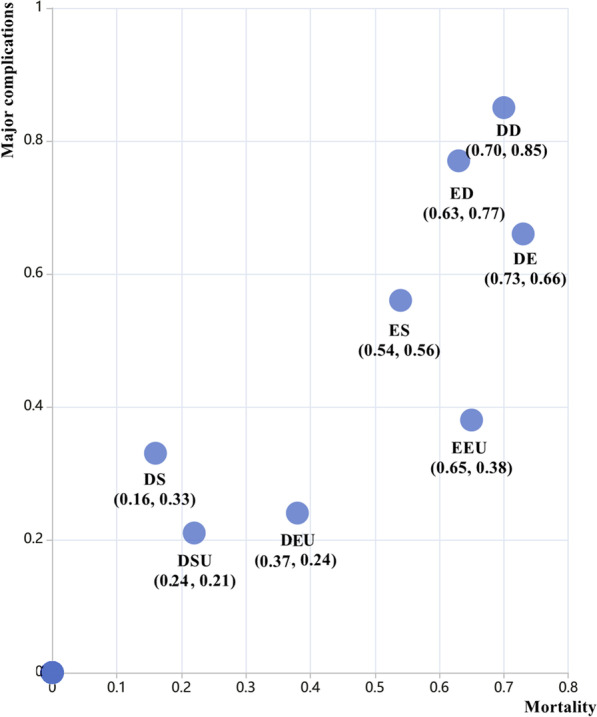
Clustered ranking plot for interventions for necrotizing pancreatitis according to mortality and major complications rankings. The horizontal axis and the vertical axis represented the SUCRA score of mortality and major complications, respectively. The SUCRA score of 1 indicated a 100% probability of ranking first and 0 indicated a 100% probability of ranking last. These are derived from the ranking for each outcome. DD, delayed drainage; DE, delayed endoscopic debridement; DEU, delayed endoscopic step-up approach; DS, delayed surgery; DSU, delayed surgical step-up approach; ED, early drainage; EEU, early endoscopic step-up approach; ES, early surgery; SUCRA, surface under the cumulative ranking

Clustered ranking plots for interventions for necrotizing pancreatitis according to the SUCRA values corresponding to the probability in percentages of each intervention for mortality and major complications were calculated and are plotted in Fig. 5 as a combination of primary endpoints. As shown in Fig. 5, DSU performed the best overall in reducing mortality and major complications, while DD performed the worst. In addition, DS, DSU and DEU have favorable safety.

### Secondary endpoints

**Fig. 6:**
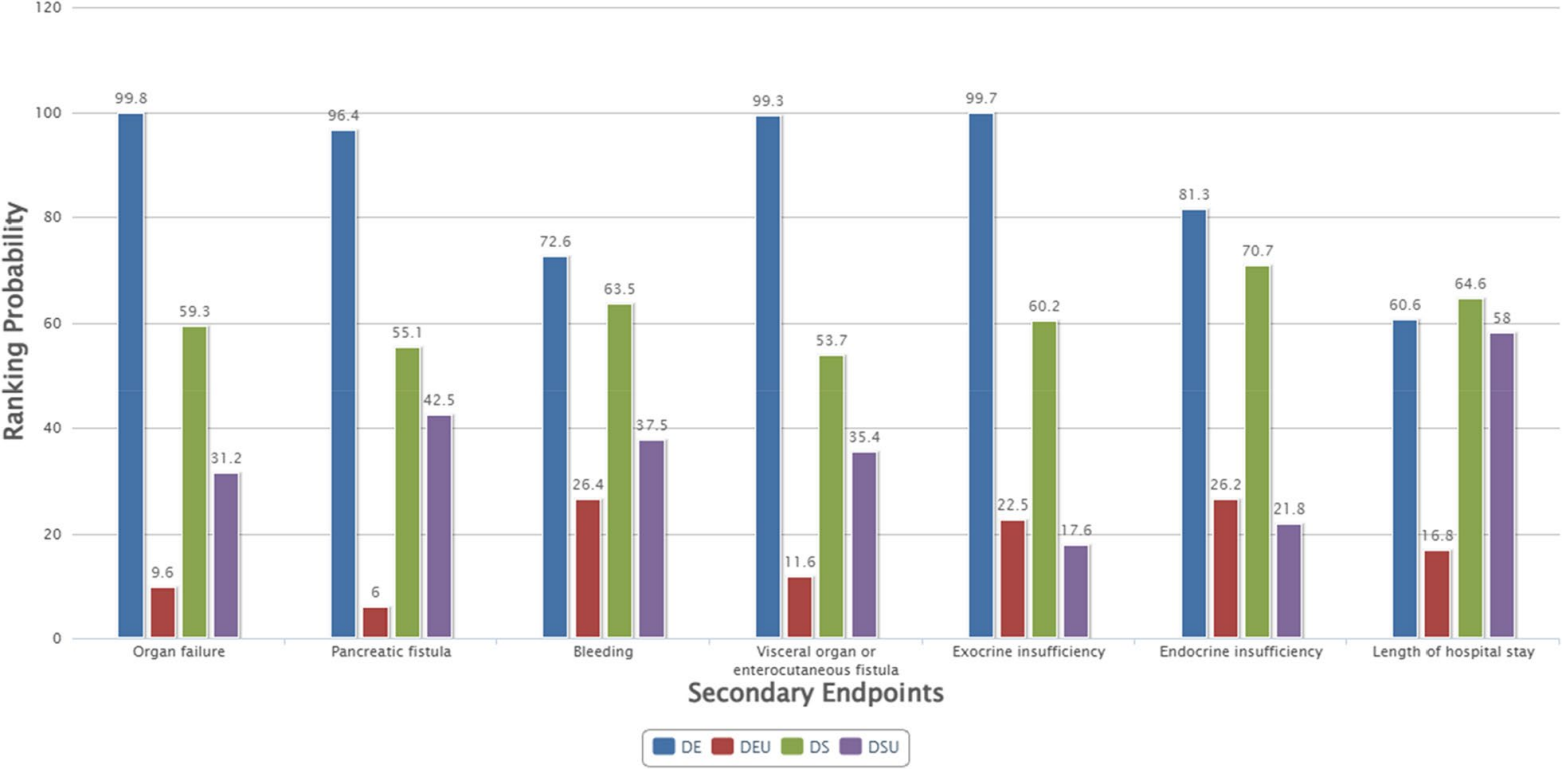
Ranking scores of DE, DS, DSU and DEU for secondary endpoints. DD, delayed drainage; DE, delayed endoscopic debridement; DEU, delayed endoscopic step-up approach; DS, delayed surgery; DSU, delayed surgical step-up approach; ED, early drainage; EEU, early endoscopic step-up approach; ES, early surgery

We assessed the secondary endpoints by comparing the 3 intervention arms, consisting of DS, DSU and DEU. Additional file 1: Appendix S6 showed direct comparisons using a traditional pairwise meta-analysis and the indirect evidence of secondary endpoints in the network meta-analysis was presented in Additional file 1: Appendix S5. Four studies [42–45] were included for bleeding, and three studies [43–45] were included for organ failure, pancreatic fistula, visceral organ or enterocutaneous fistula, exocrine insufficiency, endocrine insufficiency, and length of hospital stay. Interventions were ranked by SUCRA scores (Fig. 6). For major complications, organ failure, pancreatic fistula, bleeding and visceral organ or enterocutaneous fistula, there were similar ranking results. Participants undergoing DE had the highest probability of major complications: organ failure (99.8%), pancreatic fistula (96.4%), bleeding (71.8%) and visceral organ or enterocutaneous fistula (99.3%); in contrast, DEU was the least likely (9.6%, 6.0%, 24.6% and 11.6%, respectively). For exocrine insufficiency and endocrine insufficiency, DE was at the greatest risk (99.7% and 81.3%, respectively), while DEU and DSU had similar probabilities (22.5% vs 17.6% and 26.2% vs 21.8%, respectively). Regarding the length of hospital stay, patients with DE were the most likely to have a longer hospital stay (60.6%); in contrast, DEU was superior to the other interventions (16.8%).

### Inconsistency, heterogeneity and transitivity assessment

Node-splitting consistency models revealed no evidence of inconsistency in the networks devised for mortality and major complications (Additional file 1: Appendix S8); however, due to the absence of a closed loop in the network, node splitting was unable to be performed on the outcomes consisting of organ failure, pancreatic fistula, bleeding and visceral organ or enterocutaneous fistula, exocrine insufficiency, endocrine insufficiency and length of hospital stay.

The convergence of the calculated model was estimated using trace plots and Brooks–Gelman–Rubin diagnostic method to reveal the stability and replicability of the inferential iterations for each Markov Chain Monte Carlo chain (Additional file 1: Appendix S9). No significant heterogeneity was detected for the primary endpoints (both *I*^2^ < 50%) (Additional file 1: Appendix S10).

We conducted a network meta-regression analysis for mortality to evaluate similarity and transitivity within the included trials. The covariates analyzed were sample size, country, publication year, gender ratio, mean age, presence of organ failure and extent of necrosis ≥ 30%. The final association was calculated based on the B value and 95% CI. According to the meta-regression results described in Additional file 1: Appendix S11, the clinical characteristics did not affect the final results of mortality in this study, which implies that the transitivity was acceptable.

### Reporting bias and certainty assessment

Regarding mortality, a funnel plot with Egger’s test of the included studies did not find any significant publication bias (*p* = 0.144) (Additional file 1: Appendix S12).

We used CINeMA methods to assess the certainty of evidence. The overall confidence rating of evidence for mortality was “low” to “moderate” (Additional file 1: Appendix S13). This was due to the low risk of within-study bias, low to moderate risk of indirectness, low to some concerns of heterogeneity, low to major concerns of imprecisions, low risk of reporting bias, and low risk of incoherence in findings.

## Discussion

The present network meta-analysis compared the outcomes of 8 interventions for necrotizing pancreatitis across 10 studies with a total of 816 patients. We identified the optimal treatment strategy for necrotizing pancreatitis with low possibilities of mortality and major complications as DSU. Our findings suggested that postponement strategy interventions and step-up approach, which were usually selected in the current clinical practice, were reasonable.

Several meta-analyses have attempted to find the optimal timing and superior interventions for necrotizing pancreatitis through comparisons between two treatment strategies and concluded that the endoscopic approach had a potential advantage of a lower major complication rate over open surgery and that the timing of intervention was a risk factor for adverse outcomes [12, 24, 25]. However, they were limited by the availability of pairwise comparisons between interventions. The present NMA was conducted to compare multiple interventions simultaneously through Bayesian modeling while maintaining the randomization and assessing the relative benefit of each intervention arm, which provided more comprehensive results than precious pairwise comparisons.

In 2020, Claudio R. et al. conducted a network analysis using a frequentist method comparing early surgical debridement, peritoneal lavage, delayed surgical debridement, a step-up approach with minimally invasive debridement and a step-up approach with endoscopic debridement across 7 RCTs from 1984 to 2019 [27]. They found that a step-up approach with endoscopic debridement was the first choice for suspected infected pancreatic necrosis (IPN). However, they ignored the timing of step-up approaches and combined different interventions into a single category, which could affect outcomes. Our study included more comprehensive literature than the previous article from 1984 to 2022, ensured that the participants of the included studies could randomize into any of the interventions and avoided combining interventions into a single category by carrying out more detailed comparisons. Based on these findings, our study provided the most comprehensive evidence base currently available to guide the choices of intervention strategies for patients with necrotizing pancreatitis.

In the present study, the SUCRA scores indicated that patients who underwent early interventions (within 72 h), consisting of early drainage (ED), early surgery (ES) and early endoscopic step-up approach (EEU), all had high possibilities of major complications and mortality. Our findings suggested that delayed interventions were reasonable in terms of safety and effectiveness. Some necrotizing pancreatitis can resolve without intervention and the delayed approach can avoid unnecessary interventions [50]. Patients with early intervention may receive more procedures for necrotizing pancreatitis, and these more frequent procedures would in turn increase the risk of major complications, even leading to high mortality rates. Previous studies have confirmed that the postponement strategy averted the need for intervention in a notable proportion (40%) of patients assigned to the delayed intervention group for necrotizing pancreatitis [51]. Furthermore, as stated in the revised Atlanta classification, the delayed approach for necrotizing pancreatitis promoted a complete encapsulation of ANCs in 4 weeks, making it safer with restricted diffusion of pancreatic necrotic tissue [5, 7].

Conservative therapy for necrotizing pancreatitis included fluid resuscitation, analgesic, combination antimicrobials and nutritional support, with or without drainage of the infected fluid collections [52–54]. Although drainage was a form of invasive intervention, it recommended that pancreatic debridement and necrosectomy should be avoided whenever possible [54]. Several studies showed that conservative therapy could be successful for necrotizing pancreatitis in contrast to previous guidelines, which considered that pancreatic debridement and necrosectomy were better [55–58]. However, our results differed from the previous studies. In our study, it was worth noting that the performance of ED and DD was poor with high possibilities of mortality and major complication rates through indirect comparison using the network meta-analysis method. It was reasonable that drainage without pancreatic debridement or necrosectomy for necrotizing pancreatitis should be avoided. This inconsistent result might be explained by the fact that there was no randomized controlled trial to compare conservative treatment with the accepted standard management for patients with necrotizing pancreatitis following the same protocol. The variable severity of illness of the patients treated at different centers, variable time of referral of patients to these hospitals, and variability in the treatment could potentially lead to bias. Thus, it was not sufficient to recommend a change in practice guidelines.

Traditional pancreatic debridement and necrosectomy was performed with open necrosectomy with extensive debridement and postoperative drainage. This invasive approach was associated with high rates of complications (34 to 95%) and mortality (11 to 39%) [59]. As an alternative to open surgery, the step-up approach aiming to provide source control, rather than complete removal of the pancreatic necrotic, was advocated as the standard treatment of necrotizing pancreatitis. In our study, the top three interventions ranked by mortality were DS, DSU and DEU, while DSU, DEU and DS ranked among the top three interventions in terms of a composite of major complications. Overall, the step-up approach reduced the possibilities of mortality or major complications. This could be as a result of reduced surgical trauma, consisting of tissue damage and proinflammatory response in patients with necrotizing pancreatitis [60]. We further explored the secondary endpoints between DS, DSU, and DEU using another meta-analysis, consisting of individual components of major complications (organ failure, pancreatic fistula, bleeding and visceral organ or enterocutaneous fistula), length of hospital stay, exocrine insufficiency and endocrine insufficiency; DS had the highest probability of being ranked first. In other words, the step-up approach DEU and DSU performed better. The hypothesis is that the step-up approach could reduce tissue damage and proinflammatory response was supported by the lower incidence of multiple organ failure. For exocrine insufficiency and endocrine insufficiency, it might be a result of the removal of too much pancreatic parenchyma through surgery. Considering the above results, DEU and DSU were most likely to be the optimal treatment strategy.

Compared to the surgical step-up approach, it was considered that the endoscopic step-up approach was a potentially less invasive alternative. As the previous study reported, the endoscopic step-up approach had several advantages in reducing complications [61]. However, our results were inconsistent with the previous study. In the case of DSU, an effective balance between efficacy and safety was achieved, and the treatment ranked first from the bottom for mortality and second from the bottom for major complications. The results might probably result in a shift to the endoscopic step-up approach as treatment preference. Considering the primary endpoints, DSU seemed to be the optimal treatment strategy. We further compared DSU with DEU in terms of secondary endpoints. Patients with necrotizing pancreatitis who underwent DEU had a shorter hospital stay, but were more likely to suffer from exocrine insufficiency and endocrine insufficiency. The favorable results were explained by the absence of general anesthesia and reduction in surgical trauma, while the higher occurrence of unfavorable results might be because the endoscopic approach needed mastery of endoscopic skills and could cause pancreatic tissue damage unintentionally. In addition, Patients with DEU had a lower risk of pancreaticocutaneous fistulas. This finding was also supported by the previous study [45, 62].

The following conclusions can be drawn: (1) avoiding conservative therapy; (2) postponement strategy; (3) step-up approach; and (4) DSU was preferred over DEU.

The key assumption of an NMA was transitivity. The transitivity equation has the potential to make indirect comparisons. We ensured that studies included in our network meta-analysis were comparable and that participants included could be randomly assigned to any of the interventions. We conducted a network meta-regression analysis to evaluate similarity and transitivity within the included trial and obtained results that clinical characteristics did not affect the final results of mortality in this study. The direct and indirect evidence were consistent through the assessment for inconsistency using the node-splitting method. In addition, no obvious heterogeneity was detected among our studies. The results presented above indicated that transitivity was acceptable.

Based on the above results and discussion, the confidence rating of certainty assessment in the network analysis using the CINeMA program was “low” to “moderate.” This was due to the low risk of within-study bias, low to moderate risk of indirectness, low to major concerns of heterogeneity, low risk of reporting bias, and low risk of incoherence in findings. The low confidence might be caused by the results that imprecisions of DD and EEU comparison and indirectness of DS and EEU comparison were rated as “Major concerns.”

This NMA has several limitations. First, a limited number of RCTs were included, with a relatively low total number of participants assigned to individual groups. This might potentially explain the lack of statistically significant NMA outputs. In addition, compared to pairwise meta-analysis, network meta-analysis improved precision by adding indirect evidence to direct evidence. This treatment effect corresponded to any beneficial effect. Thus, even a small difference was considered important, leading to one treatment being preferred over another. Second, the time span of study inclusion was long, ranging from 1984 to 2021, during which time endoscopic and laparoscopic techniques developed rapidly. Technique advances seemed to have further improved the treatment outcomes of necrotizing pancreatitis in terms of mortality, major complications and length of hospital stay. Third, there was no consensus about the definitions of major complications of necrotizing pancreatitis, which might have affected the results. Patients not meeting the definition of primary and secondary endpoints in Additional file 1: Appendix S2 were excluded. Therefore, the possibility of misclassification could not be excluded. Finally, overemphasis on the ranking probability might be misleading, as it only confirmed trends in the NMA outputs. More RCTs are needed to confirm the direct and indirect evidence from network analysis.

In conclusion, DSU was the optimal treatment strategy for necrotizing pancreatitis. Drainage alone should be avoided in clinical practice. Any interventions should be postponed for at least 4 weeks if possible. The step-up approach was preferred. These findings may assist in guiding clinicians in choosing the optimal treatment strategy in clinical practice.

### Registration and protocol

This review was registered prospectively in PROSPERO (CRD42022364409).

## Supplementary Information

**Additional file 1: Appendix S1.** Detailed search strategy. **Appendix S2.** Definitions of primary and secondary endpoints. **Appendix S3.** The detailed baseline characteristics of included trials within the network meta-analysis. **Appendix S4.** Outcomes of network meta-analysis reported in included trials. **Appendix S5.** Indirect evidence of secondary endpoints in the Bayesian network meta-analysis. **Appendix S6.** Direct evidence of pairwise meta-analysis. **Appendix S7.** Network of included trials comparing interventions for necrotizing pancreatitis. **Appendix S8.** Inconsistency Analysis of Network Meta-analysis Results. **Appendix S9.** Convergence of the three Markov Chain Monte Carlo (MCMC) chains established by the history feature for mortality and major complications. **Appendix S10.** Heterogeneity Analysis of Network Meta-analysis Results. **Appendix S11.** Potential effect modifiers examined by network meta-regression models. **Appendix S12.** Adjusted funnels of mortality assessing for reporting bias. **Appendix S13.** GRADE ratings for the network using the CINeMA (Confidence in Network Meta-Analysis) program.

## Data Availability

All available data are provided in the manuscript and additional files.

## Abbreviations

ANC: Acute necrotic collections
AP: Acute pancreatitis
APACHE II: Acute Physiology and Chronic Health Evaluation II
CINeMA: Confidence in Network Meta-analysis
CIs: Confidence intervals
CrI: Credible interval
DD: Delayed drainage
DE: Delayed endoscopic debridement
DEU: Delayed endoscopic step-up approach
DS: Delayed surgery
DSU: Delayed surgical step-up approach
ED: Early drainage
EEU: Early endoscopic step-up approach
ES: Early surgery
GRADE: Grading of Recommendations Assessment, Development, and Evaluation
IPN: Infected pancreatic necrosis
MD: Mean difference
NMA: Network meta-analysis
NP: Necrotizing pancreatitis
ORs: Odds ratios
PICOS: Population, Intervention, Comparator, Outcome and Study design
PRISMA: Preferred Reporting Items for Systematic Reviews and Meta-Analyses
RCTs: Randomized controlled trials
SD: Standard deviation
SUCRA: Surface under the cumulative ranking
VARD: Video-assisted retroperitoneal debridement
WON: Walled-off necrosis
