# Supplementary material for: The optimal timing and intervention to reduce mortality for necrotizing pancreatitis: a systematic review and network meta-analysis
Source: World J Emerg Surg. 2023 Jan 27;18:9. doi: 10.1186/s13017-023-00479-7 (PMC9883927; doi:10.1186/s13017-023-00479-7)
Supplement: Supplementary file 1 — Additional file 1: Appendix S1. Detailed search strategy. Appendix S2. Definitions of primary and secondary endpoints. Appendix S3. The detailed baseline characteristics of included trials within the network meta-analysis. Appendix S4. Outcomes of network meta-analysis reported in included trials. Appendix S5. Indirect evidence of secondary endpoints in the Bayesian network meta-analysis. Appendix S6. Direct evidence of pairwise meta-analysis. Appendix S7. Network of included trials comparing interventions for necrotizing pancreatitis. Appendix S8. Inconsistency Analysis of Network Meta-analysis Results. Appendix S9. Convergence of the three Markov Chain Monte Carlo (MCMC) chains established by the history feature for mortality and major complications. Appendix S10. Heterogeneity Analysis of Network Meta-analysis Results. Appendix S11. Potential effect modifiers examined by network meta-regression models. Appendix S12. Adjusted funnels of mortality assessing for reporting bias. Appendix S13. GRADE ratings for the network using the CINeMA (Confidence in Network Meta-Analysis) program. [file 13017_2023_479_MOESM1_ESM.docx]

**Appendix S1. Detailed search strategy**

Till 30 November 2022

| Database | Search terms and limitations | |
| --- | --- | --- |
| Pubmed  (n=226) | #1 | Search ((necrotizing pancreatitis[MeSH Terms]) OR (pancreatitis[MeSH Terms]) OR (Necrotizing Pancreatitis, Acute[Title/Abstract]) OR (Pancreatitis Necrotising[Title/Abstract])) |
|  | #2 | Search ((drainage[Title/Abstract]) OR (surgery[Title/Abstract]) OR (endoscopic[Title/Abstract]) OR (step-up approach[Title/Abstract]) OR (debridement[Title/Abstract]) OR (endoscopy[Title/Abstract])) OR (surgical[Title/Abstract])) |
|  | #3 | #1 AND #2 |
| Embase  (n=990) | #1 | ('pancreatitis necrotising'/exp OR 'severe pancreatitis':ti,ab,kw OR 'acute hemorrhagic pancreatitis':ti,ab,kw OR 'necrotizing pancreatitis':ti,ab,kw) |
|  | #2 | MeSH descriptor: [pancreatitis necrotizing] explode all trees |
|  | #3 | MeSH descriptor: [pancreatic necrosis] explode all trees |
|  | #4 | ('drainage'/exp OR drainage OR surgery:ti,ab,kw OR endoscopy:ti,ab,kw OR 'step-up approach':ti,ab,kw OR debridement:ti,ab,kw OR surgical:ti,ab,kw OR endoscopic:ti,ab,kw) |
|  | #5 | (#1 OR #2 OR #3) AND #4 |
| Cochrane library  (n=804) | #1 | (pancreatitis necrotizing*:ab,ti ) OR ( necrotizing pancreatitis :ab,ti) OR ( pancreatic necrosis :ab,ti) |
| ClinicalTrials.gov  (n=5) | #1 | (Necrotizing pancreatitis OR Pancreatic Necrosis) with results |

**Appendix S2 Definitions of primary and secondary endpoints**

Mortality: death during admission and until 6 months after discharge

Major complications: a composite of new-onset multiple organ failure or systemic dysfunction, enteral or pancreatic-cutaneous fistula, bleeding, visceral perforation, or death during admission and until 6 months after discharge.

Organ failure: pulmonary failure, circulatory failure, renal Failure and multiple organ failure

Pancreatic fistula: output via a percutaneous or nasocystic drain or drainage canal after removal of percutaneous drains or from a surgical wound of any measurable volume of fluid with an amylase content greater than 3 times the serum amylase activity

Bleeding: bleeding within the intraabdominal cavity or gastrointestinal tract requiring interventional procedure

Visceral organ or enterocutaneous fistula: perforation of a visceral organ requiring interventional procedure or formation of a fistula between the small bowel or colon and skin as indicated by

feculent output from a percutaneous catheter or surgical wound site, confirmed on imaging or surgery

Exocrine insufficiency: oral pancreatic-enzyme supplementation required to treat clinical

symptoms of steatorrhea 6 months after randomization; this requirement was not present before onset of pancreatitis

Endocrine insufficiency: insulin or oral antidiabetic drugs required 6 months after randomisation; this requirement was not present before onset of pancreatitis

Length of hospital stay: total number of days in hospital within 6 months

**Appendix S3 The detailed baseline characteristics of included trials within the network meta-analysis**

| Author, year | Comparision | Etiology | | | APACH II, median (IQR) | %Organ failure | %Extent of necrosis  ≥ 30% | CT severity index, median (IQR) |
| --- | --- | --- | --- | --- | --- | --- | --- | --- |
|  |  | %Biliary | %Hypertriglyceridemia | %Alcohol abuse |  |  |  |  |
| Ke,L., 2021^48^ | ED | 46.7% | 53.3% | NR | 16 (7–18) | 24% | 80.0% | NR |
|  | DD | 33.3% | 66.7% | NR | 13 (10–20) | 16% | 86.7% | NR |
| Boxhoorn, L.,2021^47^ | ED | 65% | NR | 15% | NR | 24% | 36.4.7% | 7 (6–10) |
|  | DD | 59% | NR | 14% | NR | 16% | 32.7% | 6 (6–8) |
| Bang,J., 2019^46^ | ES | 41.2% | NR | 17.6% | 30 (26–35) | 28.1% | NR | NR |
|  | EEU | 25.0% | NR | 34.4 | 21 (16–23) | 29.4% | NR | NR |
| van Brunschot, 2018^45^ | DEU | 51% | NR | 14% | 9 (5–13) | 25% | 49.0% | 6 (6–8) |
|  | DSU | 64% | NR | 15% | 10 (6–13) | 30% | 53.2% | 8 (6–10) |
| Bakker, 2012^44^ | DE | 70% | NR | 20% | 11 (7-14) | 30% | 60% | 8 (4-10) |
|  | DS | 60% | NR | 20% | 10 (6-14) | 20% | 60% | 8 (4-10) |
| van Santvoort, 2010^43^ | DS | 60% | NR | 7% | NR | 49% | 57.8% | 8 (4-10) |
|  | DSU | 64% | NR | 11% | NR | 49% | 60.5% | 8 (4-10) |
| Litvin A, 2010^42^ | DSU | NR | NR | NR | NR | NR | NR | NR |
|  | ES | NR | NR | NR | NR | NR | NR | NR |
| Mier, J., 1997^41^ | ES | 24.0% | 8.0% | 52.0% | NR | NR | NR | NR |
|  | DS | 54.5% | 9.1% | 27.3% | NR | NR | NR | NR |
| Schröder,T, 1991^40^ | ES | NR | NR | NR | NR | NR | NR | NR |
|  | ED | NR | NR | NR | NR | NR | NR | NR |
| Kivilaakso，E, 1984^39^ | ES | NR | NR | NR | NR | NR | NR | NR |
|  | ED | NR | NR | NR | NR | NR | NR | NR |

Data were derived from CT performed before patients underwent randomization. Scores on the CT Severity Index range from 0 to 10, with higher scores indicating more extensive pancreatic and peripancreatic necrosis.

APACHE II; Acute Physiology and Chronic Health Evaluation II; CT, contrast-enhanced computed tomography; DD, delayed drainage; DE, delayed endoscopic debridement; DEU, delayed endoscopic step-up approach; DS, delayed surgery; DSU, delayed surgical step-up approach; ED, early drainage; EEU, early endoscopic step-up approach; ES, early surgery; IQR, interquartile range; NR, not state; SD, standard deviation

**Appendix S4 Outcomes of network meta-analysis reported in included trials**

| Author, year | Mortality | Major complications | Organ failure | Pancreatic fistula | Bleeding | Visceral organ or enterocutaneous fistula | Exocrine insufficiency | Endocrine insufficiency | Length of hospital stay |
| --- | --- | --- | --- | --- | --- | --- | --- | --- | --- |
| Ke,L., 2021^48^ | Y | N | Y | Y | Y | Y | Y | Y | Y |
| Boxhoorn, L.,2021^47^ | Y | Y | Y | Y | Y | Y | Y | Y | Y |
| Bang,J., 2019^46^ | Y | Y | Y | Y | Y | Y | Y | Y | Y |
| van Brunschot, 2018^45^ | Y | N | Y | Y | Y | Y | Y | N | Y |
| Bakker, 2012^44^ | Y | Y | Y | Y | Y | Y | Y | Y | Y |
| van Santvoort, 2010^43^ | Y | Y | Y | Y | Y | Y | Y | Y | Y |
| Litvin A, 2010^42^ | Y | Y | N | N | Y | N | N | N | Y |
| Mier, J., 1997^41^ | Y | Y | N | N | N | N | N | N | N |
| Schröder,T, 1991^42^ | Y | Y | N | N | N | N | N | N | Y |
| Kivilaakso，E, 1984^41^ | Y | Y | N | N | N | N | N | N | Y |

Y, yes; N, no

**Appendix S5 Indirect evidence of secondary endpoints in the Bayesian network meta-analysis**

1. **Odds ratios and 95% CrIs for bleeding (lower triangle in yellow)**

| DS |  | - |  |  |
| --- | --- | --- | --- | --- |
| 6.82  (0.09, 18.27) | DEU |  |  |  |
| 0.70  (0.35, 2.21) | 0.10  (-2.84, 3.05) | DSU |  |  |
| 0.01  (0.001, 0.11) | 0.01  (0.001, 0.03) | 0.01  (0.001, 0.02) | DE |  |
| 3.53  0.04, 5.64) | 0.51  (0.01, 3.66) | 0.46  (0.02, 2.34) | 19.64  (2.79, 49.77) | ES |

1. **Mean difference and 95% CrIs for length of hospital (upper triangle in green) and odds ratios and 95% CrIs organ failure (lower triangle in yellow)**

| DS | 1.98  (0.60, 3.06) | 0.92  (0.60, 1.52) | 0.55  (0.44, 4.47) |
| --- | --- | --- | --- |
| 1.31  (0.96, 3.57) | DEU | 1.55  (0.92, 2.28) | 0.20  (0.03, 2.82) |
| 1.19  (1.02, 2.77) | 0.22  (0.01, 0.73) | DSU | 0.96  (0.36, 2.66) |
| 0.04  (0.01, 1.34) | 0.29  (-0.07,1.56) | 0.17  (0.05, 0.47) | DE |

1. **Odds ratios and 95% CrIs for pancreatic fistula (upper triangle in green) and visceral organ or enterocutaneous fistula (lower triangle in yellow)**

| DS | 2.85  (0.79, 5.04) | 1.73  (1.46, 6.75) | 3.52  (0.48, 8.11) |
| --- | --- | --- | --- |
| 1.55  (-2.01, 5.16) | DEU | 9.39  (1.16, 46.08) | 0.64  (0.11, 3.05) |
| 1.84  (0.15, 3.12) | 2.33  (0.57, 7.60) | DSU | 0.04  (0.01, 0.57) |
| 0.18  (0.01, 3.73) | 0.13  (0.03, 1.58) | 0.54  (0.21, 1.93) | DE |

1. **Odds ratios and 95% CrIs for endocrine insufficiency (upper triangle in green) and exocrine insufficiency (lower triangle in yellow)**

| DS | 1.93  (0.62, 2.37) | 2.02  (0.52, 3.05) | 0.57  (0.06, 5.49) |
| --- | --- | --- | --- |
| 1.14  (0.30, 2.52) | DEU | 2.48  (1.35, 2.86) | 1.78  (0.76, 3.78) |
| 1.18  (1.04,1.87) | 1.03  (0.33, 1.70) | DSU | 1.39  (1.03, 2.64) |
| 0.93  (0.01, 1.94) | 0.84  (0.01, 1.67) | 0.16  (0.01, 3.42) | DE |

Dichotomous values were expressed as odds ratios (95%CrI) and continuous values were expressed as mean difference (95%CrI). Each cell gives the effect of the column-defining intervention relative to the row-defining intervention. Values > 1 indicated that the intervention for necrotizing pancreatitis in the corresponding column had a higher risk of secondary endpoints than those in the corresponding rows and values < 1 indicated a lower risk.

CrI, credible interval; DE, delayed endoscopic debridement; DEU, delayed endoscopic step-up approach; DS, delayed surgery; DSU, delayed surgical step-up approach; MD, mean difference; ORs, odds ratios

**Appendix S6** **Direct evidence of pairwise meta-analysis**

|  | Length of hospital | Organ failure | Pancreatic fistula | Bleeding | Visceral organ or  enterocutaneous fistula | Exocrine insufficiency | Endocrine insufficiency |
| --- | --- | --- | --- | --- | --- | --- | --- |
| ED vs DD | 1.97  (0.97, 4.06) | 0.32  (0.10, 0.96) | 1.3  (0.39,4.35) | 0.90  (0.35.2.32) | 0.45  (0.15,1.36) | 8.09  (2.55,25.65) | 0.63  (0.25,1.55) |
| DS vs DE | 0.98  (0.41, 2.34) | 0.05  (0.01, 1.03) | 0.05  (0.01,0.56) | NA | 0.16  (0.01,3.85) | 0.10  (0.01,2.88) | 0.58  (0.07,4.56) |
| DSU vs DEU | 1.45  (0.97, 2.16) | 0.19  (0.07, 0.54) | 9.37  (1.98,44.21) | 1.25  (0.49,3.18) | 2.41  (0.67,8.61) | 0.89  (0.40,2.00) | 0.97  (0.36,2.65) |
| DSU vs DS | 0.94  (0.62, 1.42) | 0.20  (0.07, 0.60) | 0.64  (0.26,1.57) | 0.68  (0.23,1.99) | 0.57  (0.19,1.73) | 0.15  (0.04,0.57) | 0.32  (0.12,0.88) |
| ES vs DS | NA | NA | NA | NA | NA | NA | NA |
| ES vs ED | 1.25  (0.71, 2.20) | NA | NA | NA | NA | NA | NA |
| ES vs EEU | 0.48  (0.02, 2.37) | 1.66  (0.26,10.62) | 27.89  (1.55,502.77) | 8.19  (0.41,165.03) | 8.19  (0.41,165.03) | 1.21  (0.29,4.96) | 1.83  (0.57,5.89） |
| EEU vs DEU | 13.1  (5.59, 20.61) | NA | NA | NA | NA | NA | NA |
| DSU vs ES | 0.01  (0.001, 0.09) | NA | NA | 0.48  (0.13, 1.83) | NA | NA | NA |

Dichotomous values were expressed as ORs (95%CI) and continuous values were expressed as MD (95%CI).

CIs, confidence interval; DD, delayed drainage; DE, delayed endoscopic debridement; DEU, delayed endoscopic step-up approach; DS, delayed surgery; DSU, delayed surgical step-up approach; ED, early drainage; EEU, early endoscopic step-up approach; ES, early surgery; MD, mean difference; NA, not application; OR, odds ratio; vs, versus

**Appendix S7 Network of included trials comparing interventions for necrotizing pancreatitis**

1. **Bleeding**


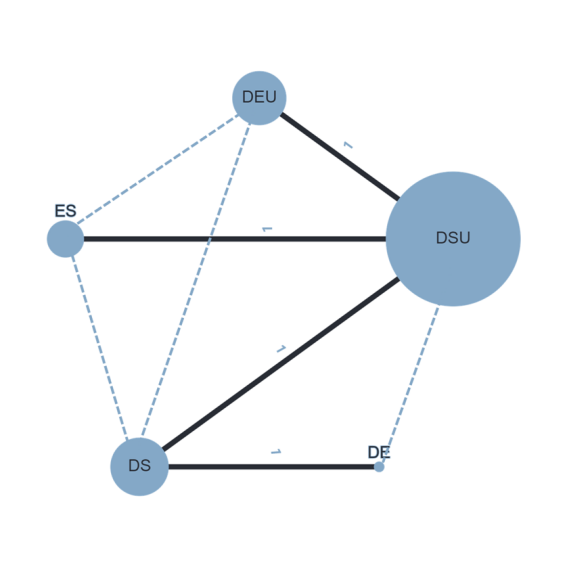


1. **Organ failure**


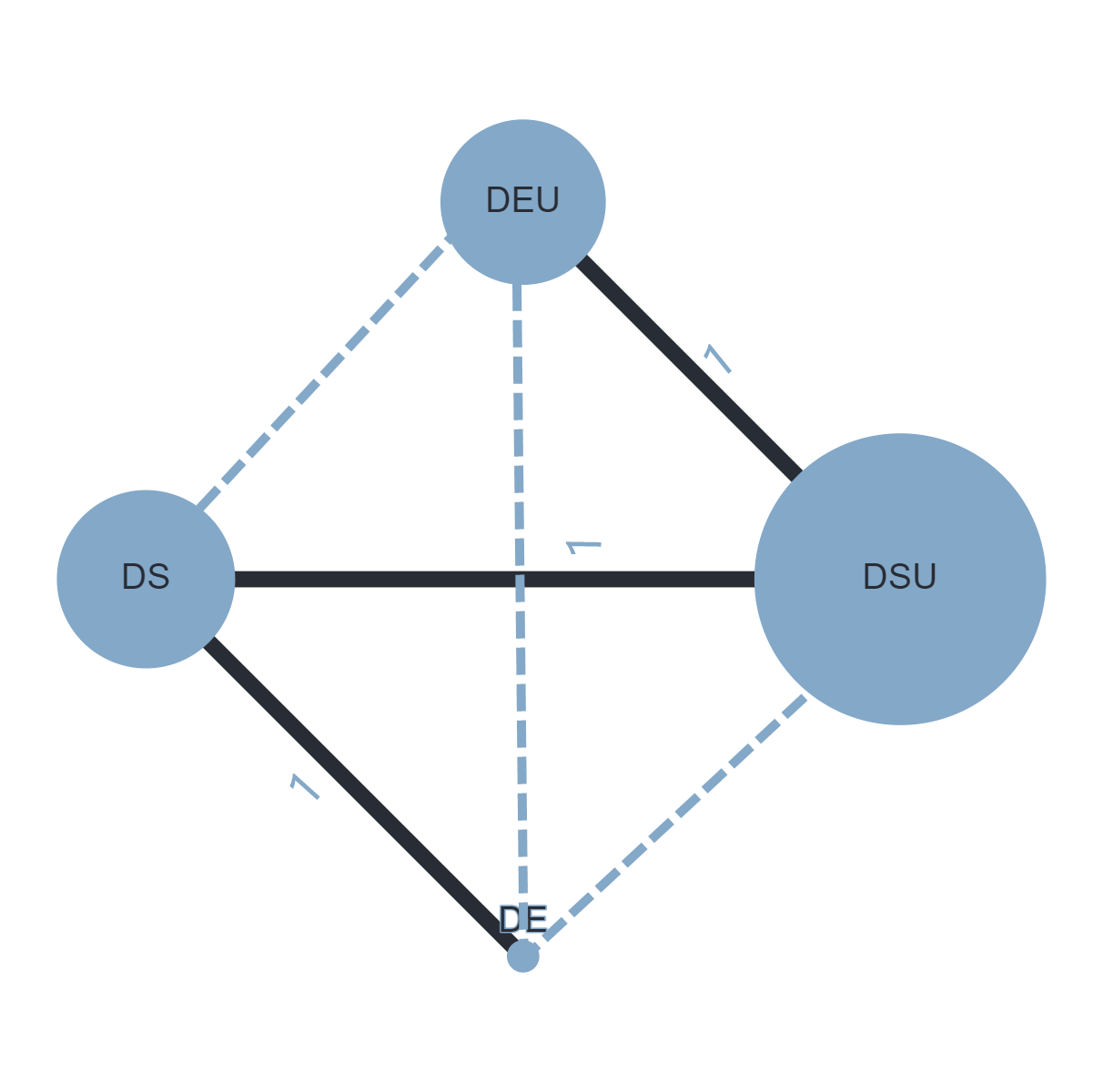


1. **Pancreatic fistula**


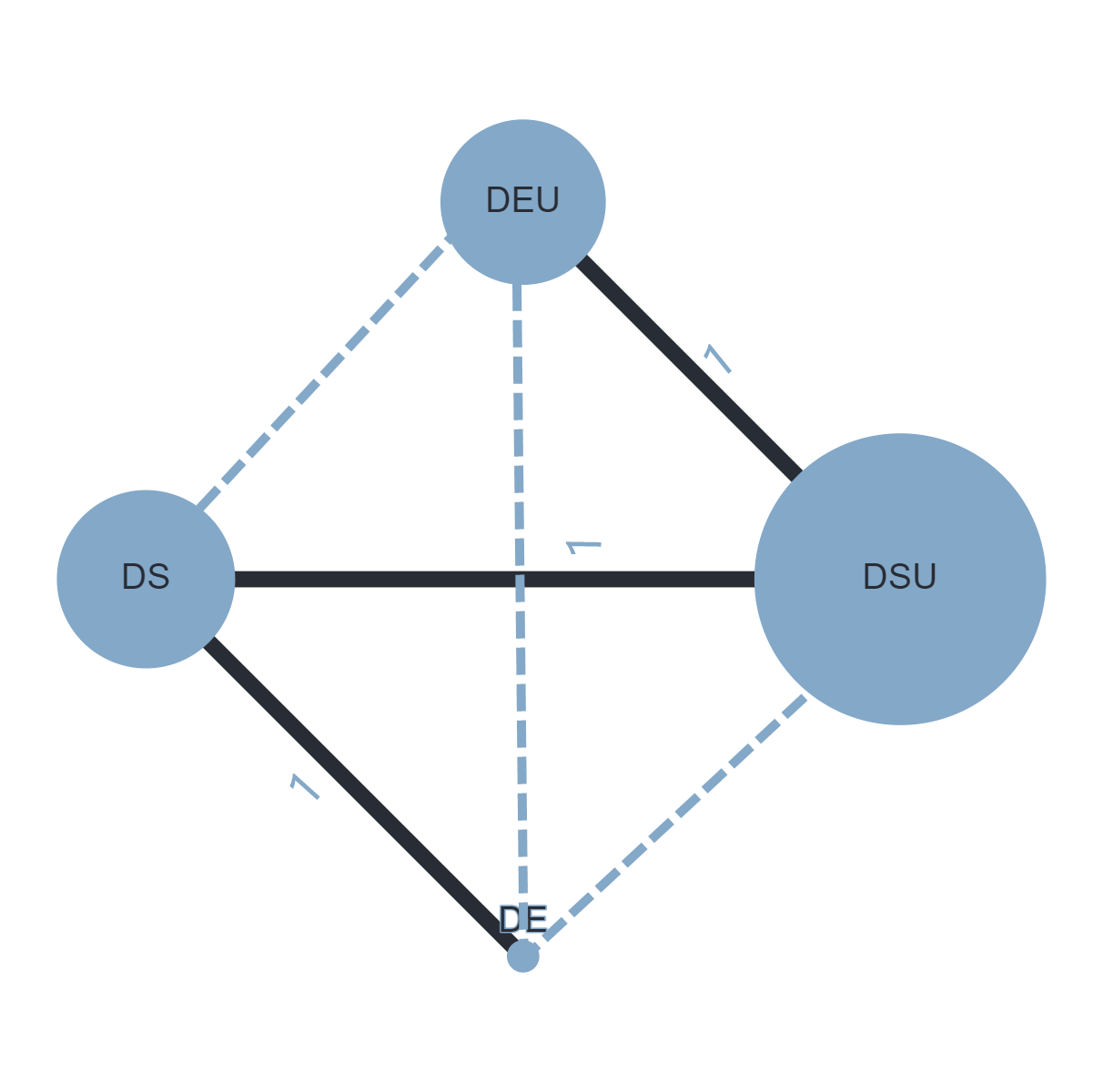


1. **Visceral organ or enterocutaneous fistula**


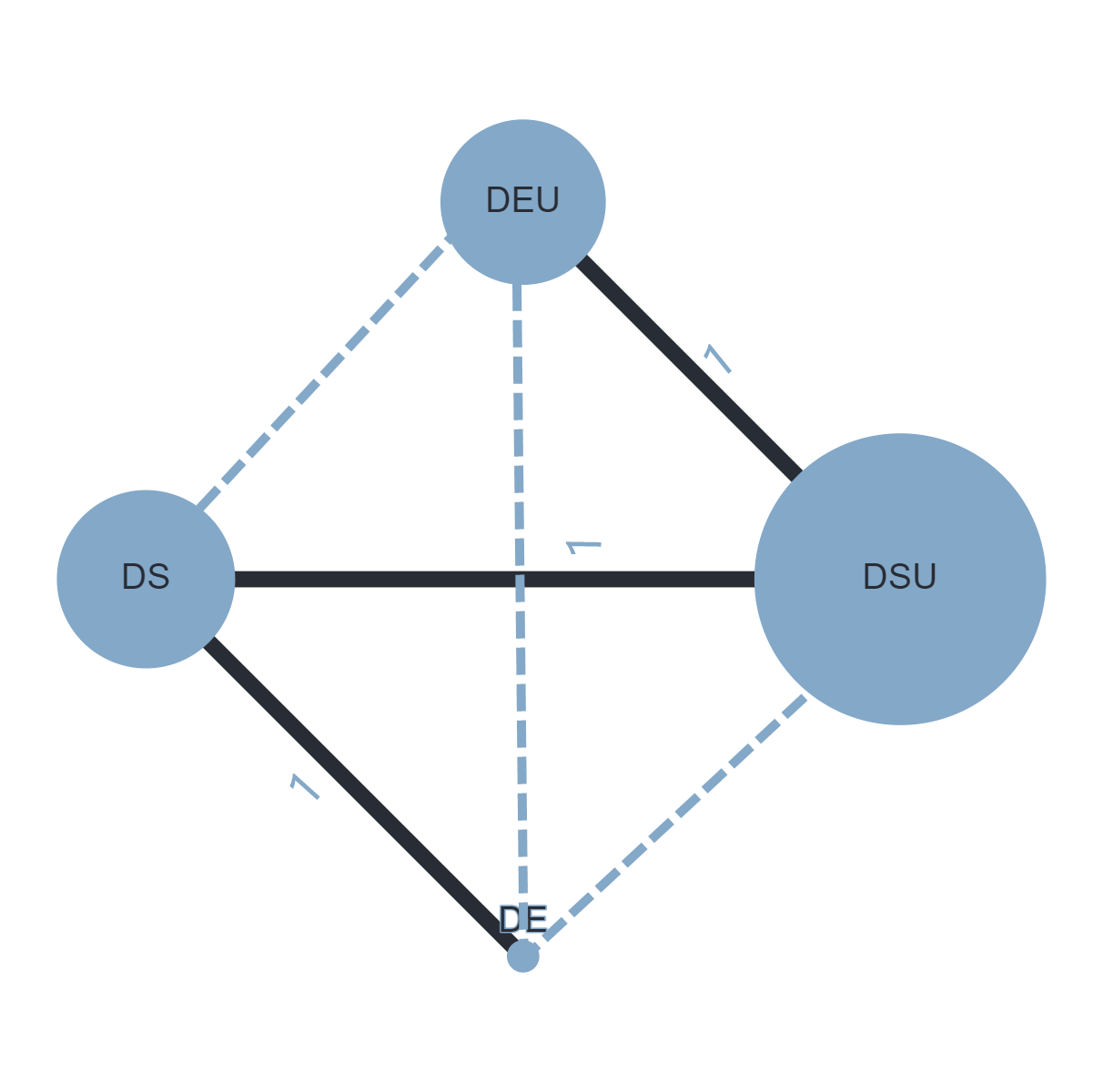


1. **Exocrine insufficiency**


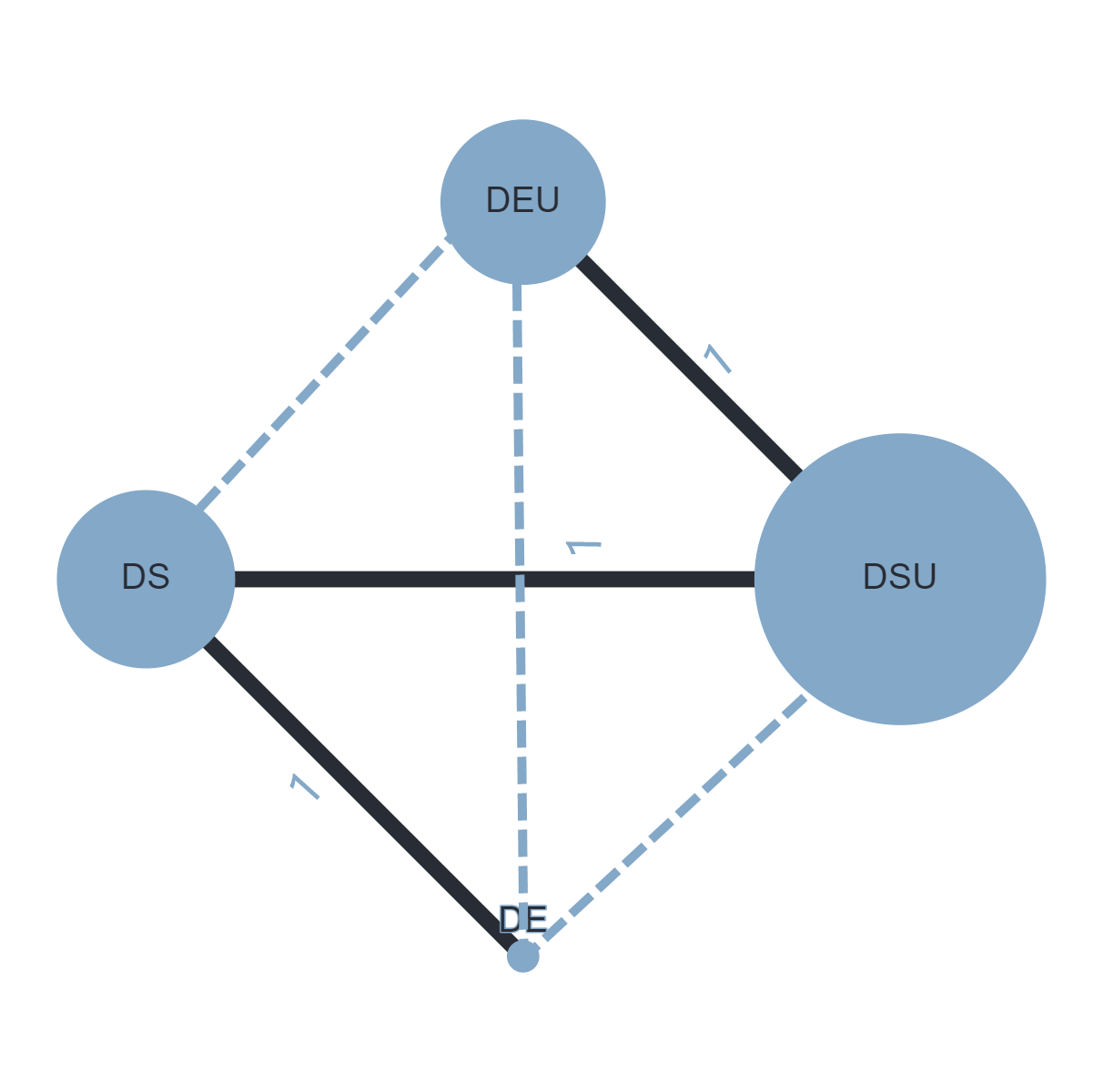


1. **Endocrine insufficiency**


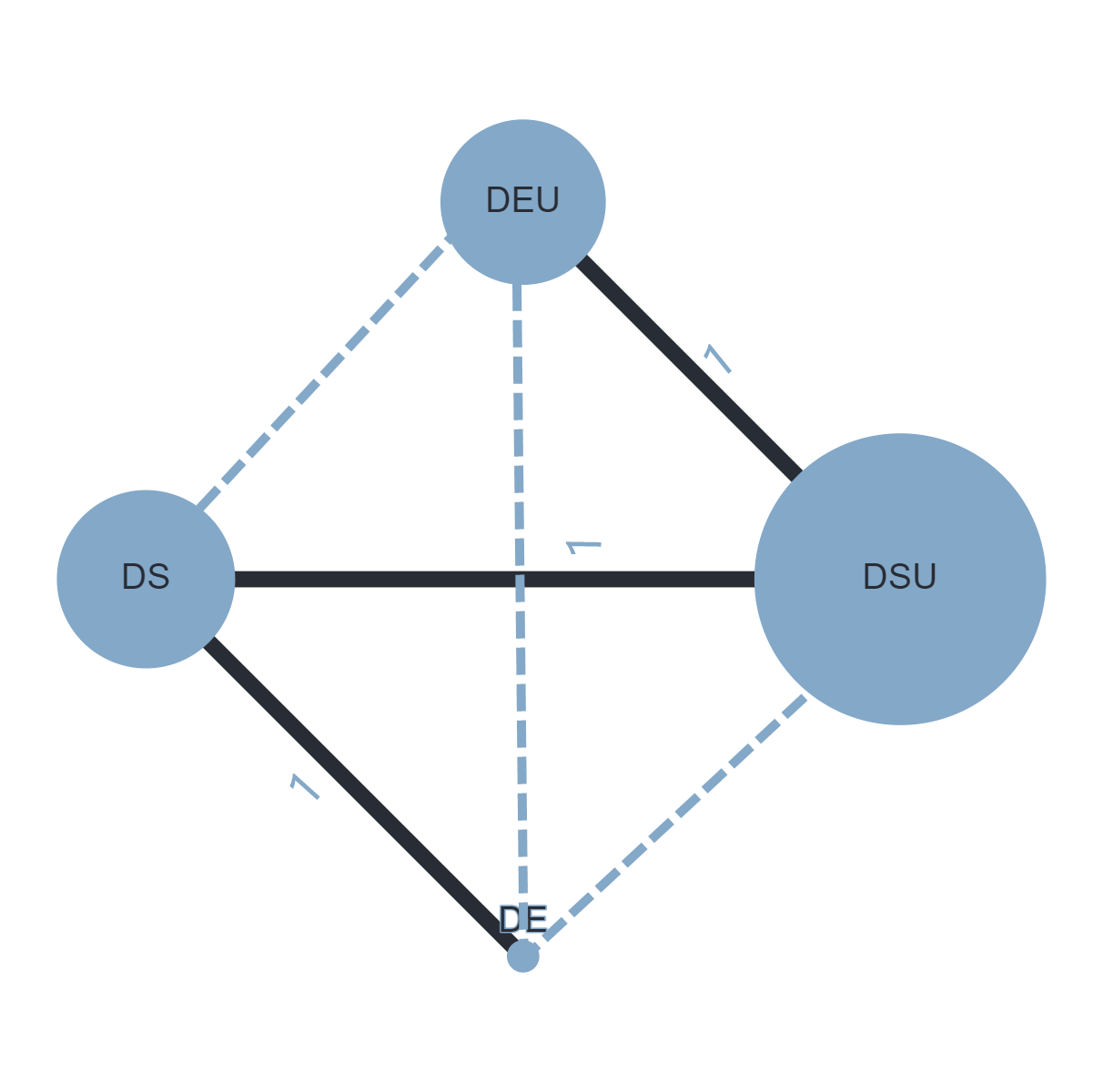


1. **Length of hospital stay**


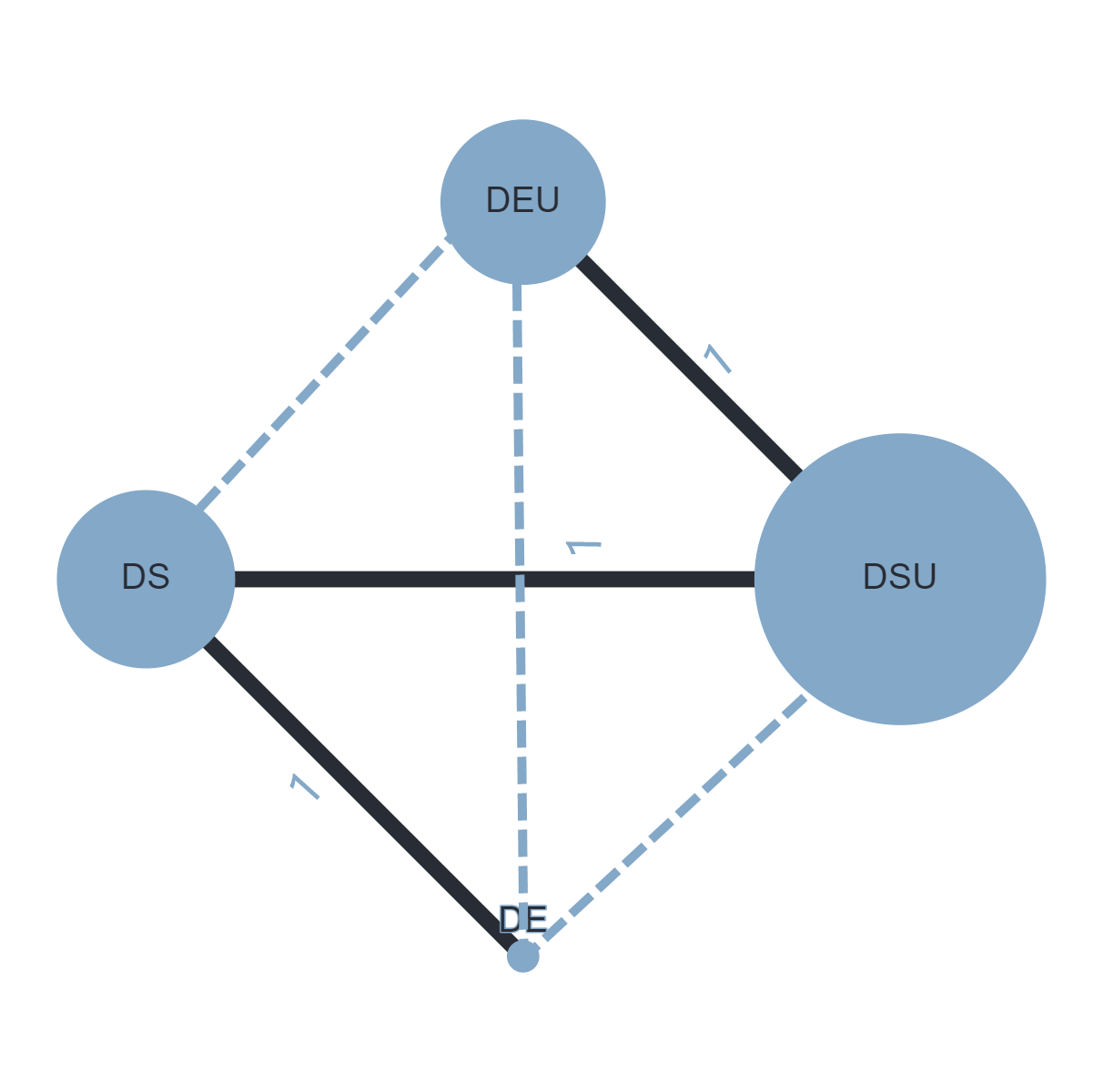


DE, delayed endoscopic debridement; DEU, delayed endoscopic step-up approach; DS, delayed surgery; DSU, delayed surgical step-up approach; ES, early surgery

**Appendix S8 Inconsistency Analysis of Network Meta-analysis Results**

1. **Mortality**


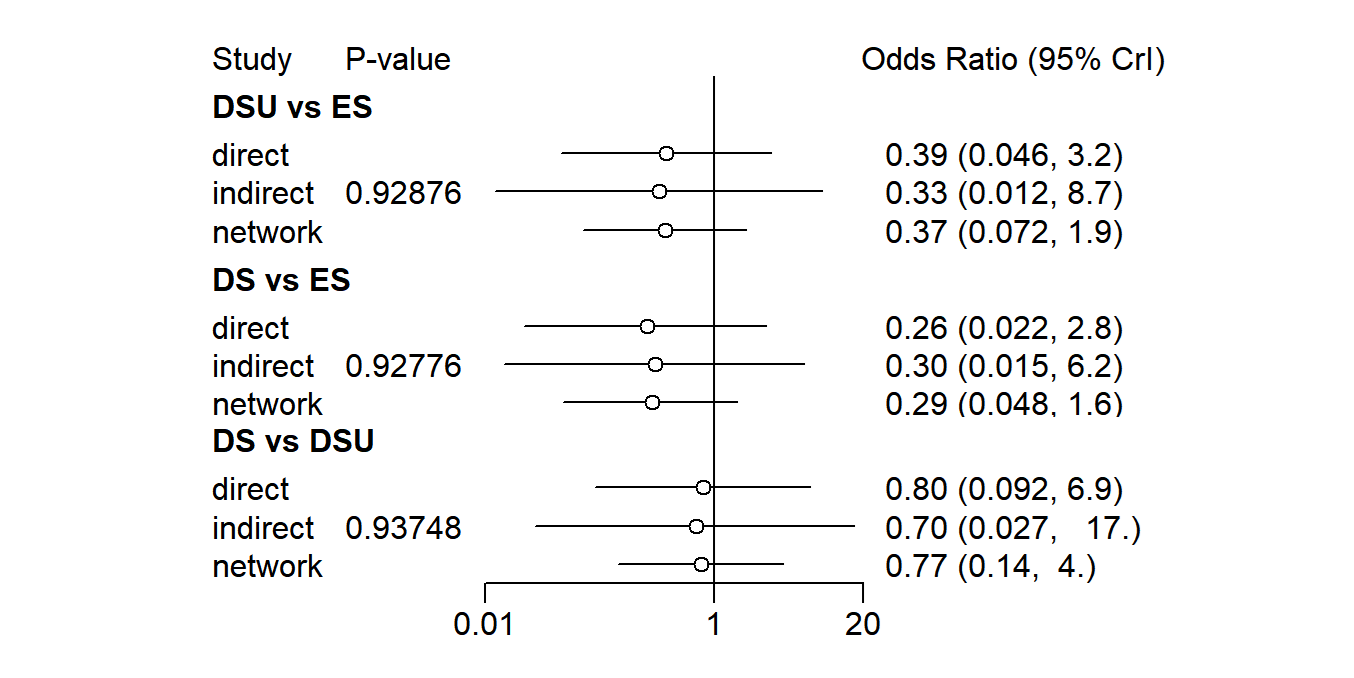


1. **Major complications**

**
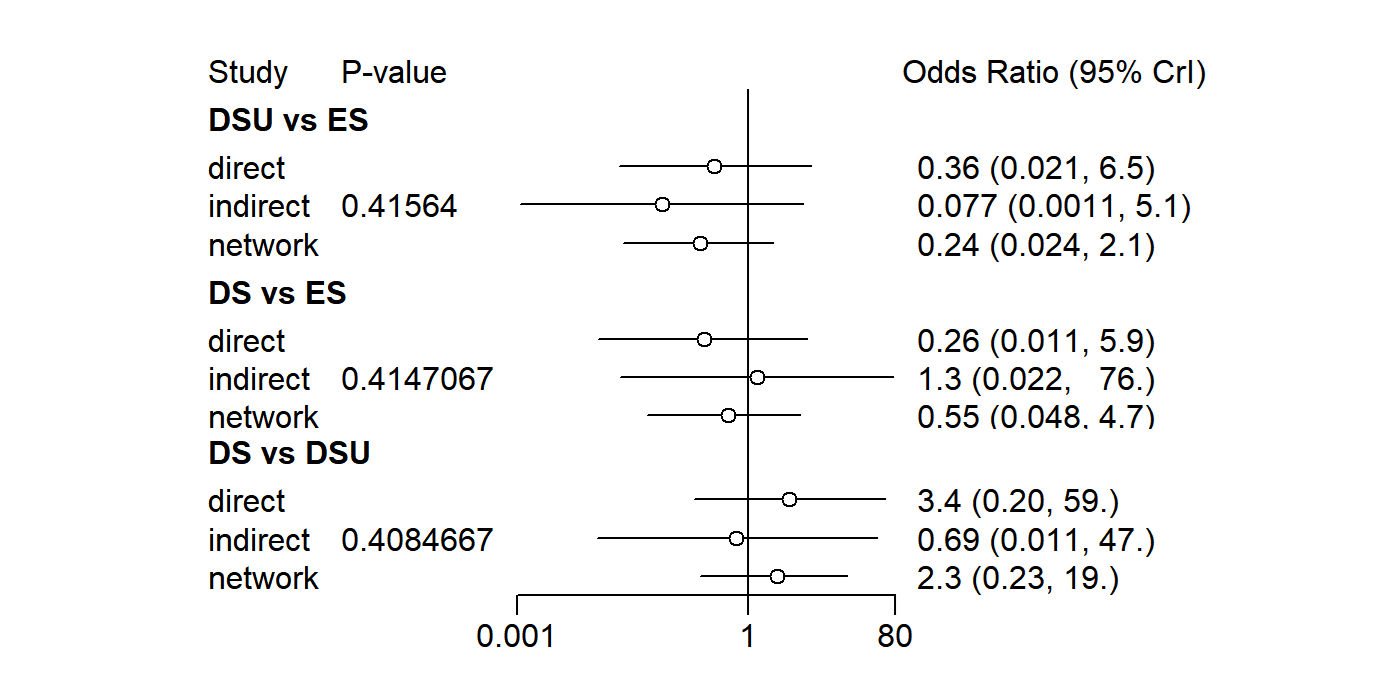
**

CrI, credible interval; DD, delayed drainage; DE, delayed endoscopic debridement; DEU, delayed endoscopic step-up approach; DS, delayed surgery; DSU, delayed surgical step-up approach; ED, early drainage; EEU, early endoscopic step-up approach; ES, early surgery

**Appendix S9** **Convergence of the three Markov Chain Monte Carlo (MCMC) chains established by of the history feature for mortality and major complications**

1. **Mortality**


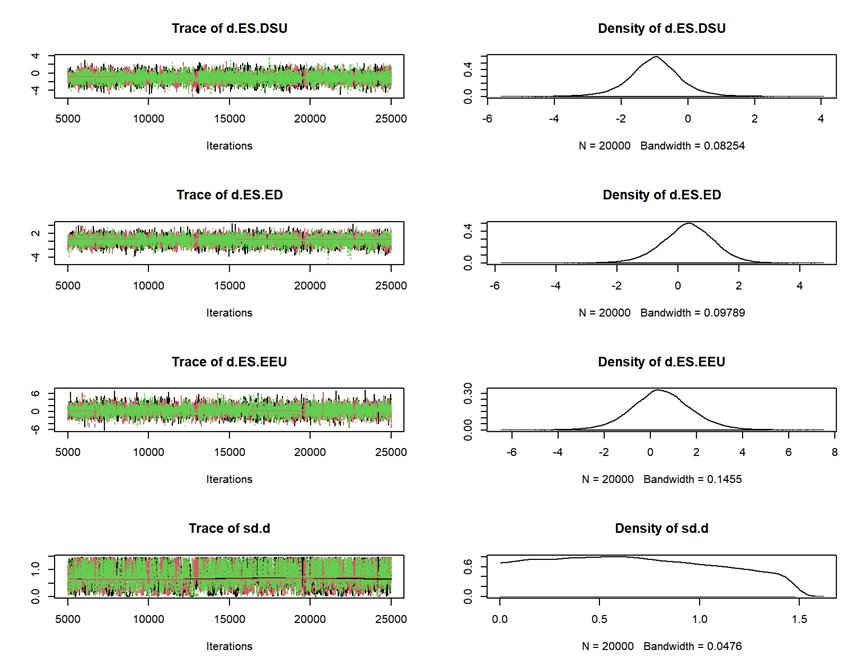


1. **Major complications**


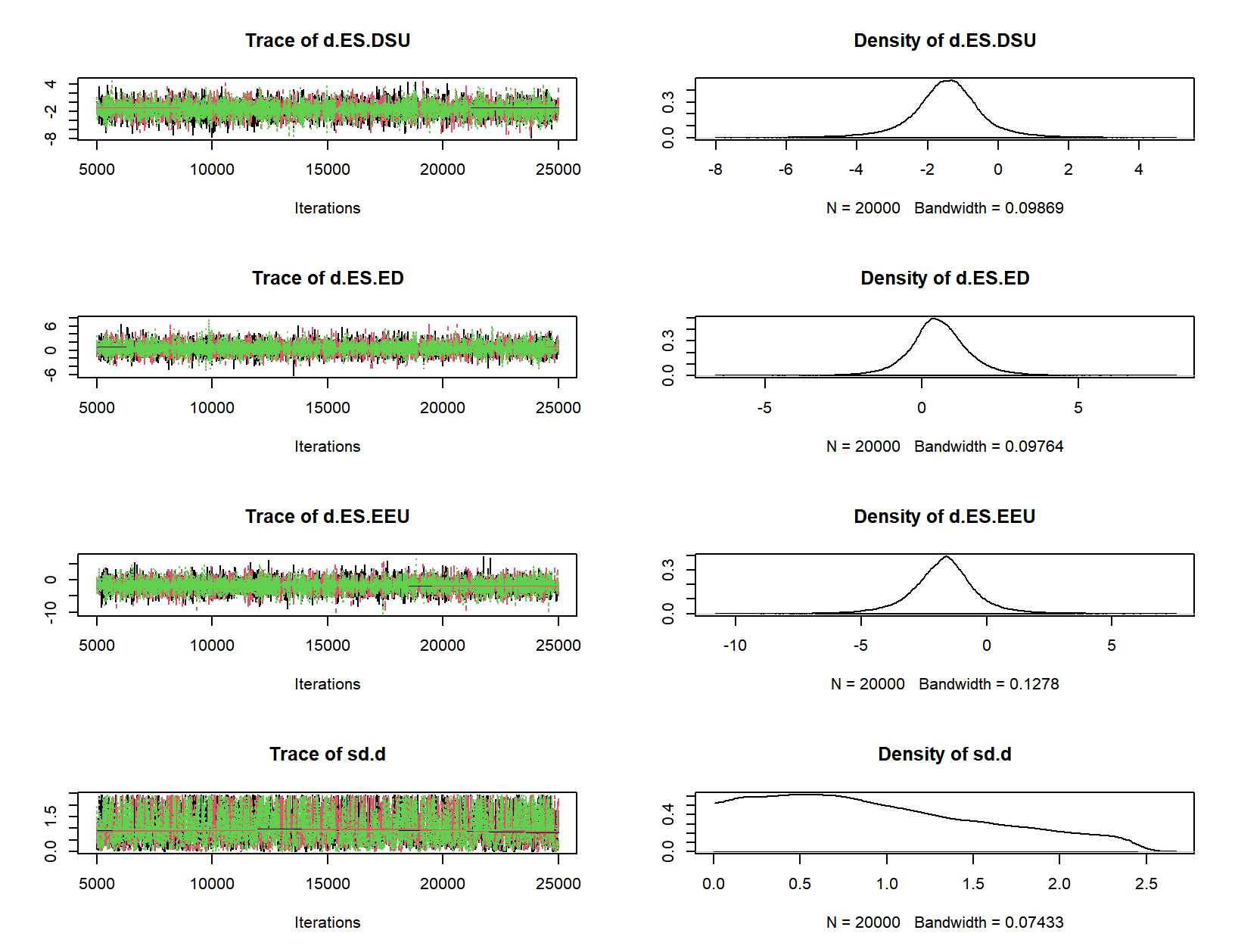


In each Bayesian inference analysis, three independent MCMC chains are generated and running 20000 sample iterations per chain simultaneously.

**Appendix S10 Heterogeneity Analysis of Network Meta-analysis Results**

1. **Mortality**

**
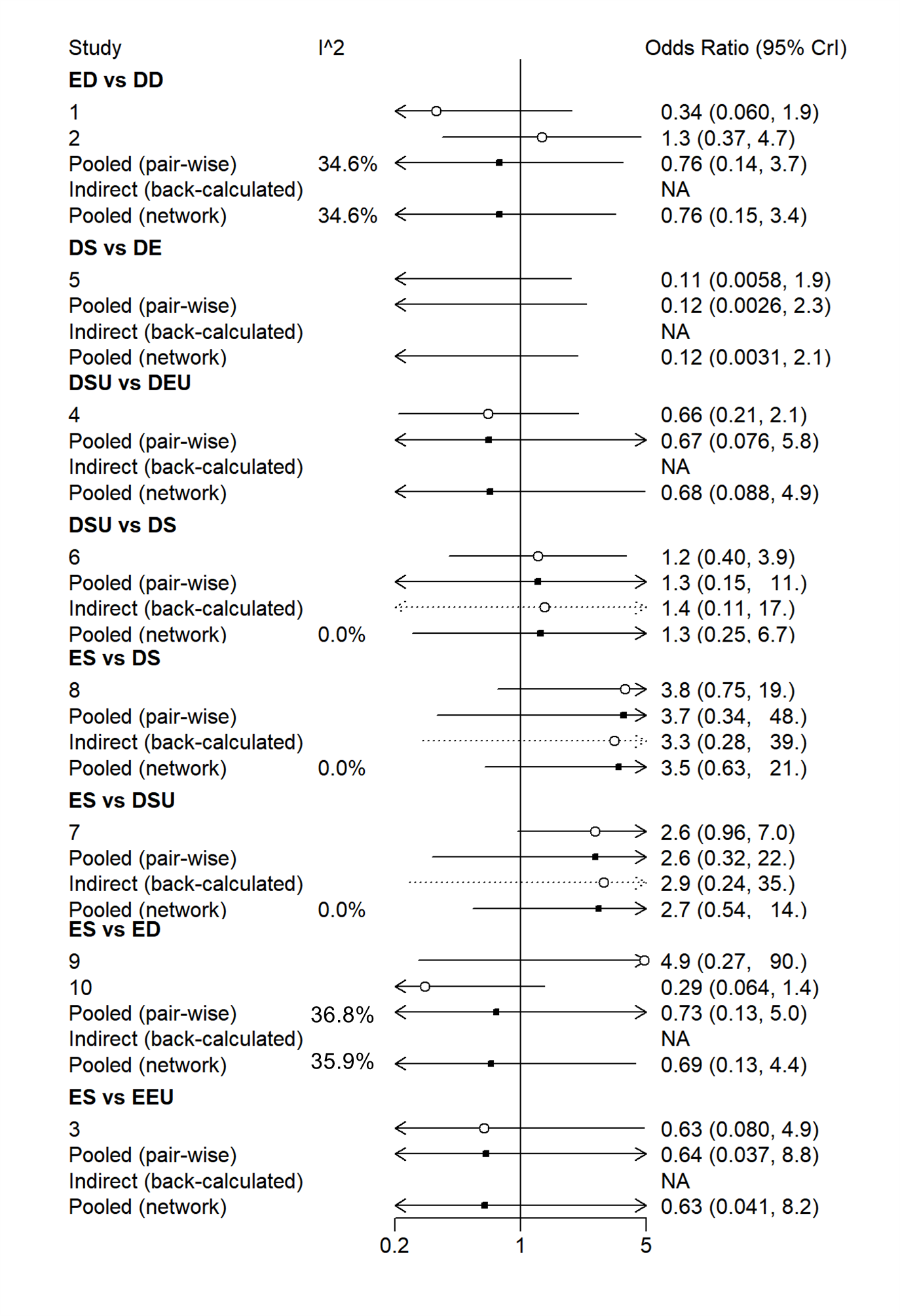
**

1. **Major complications**

**
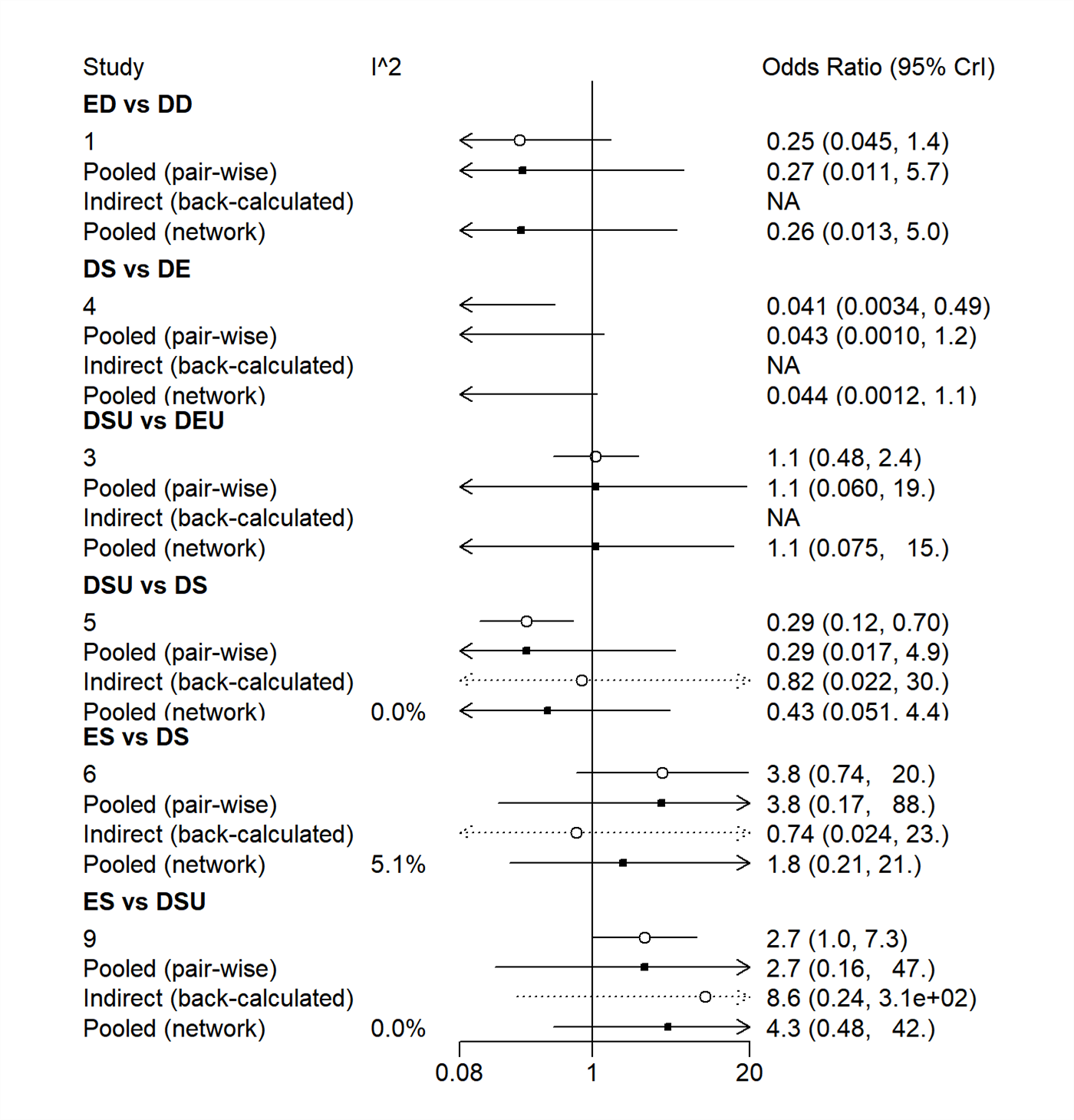
**

CrI, credible interval; DD, delayed drainage; DE, delayed endoscopic debridement; DEU, delayed endoscopic step-up approach; DS, delayed surgery; DSU, delayed surgical step-up approach; ED, early drainage; EEU, early endoscopic step-up approach; ES, early surgery

**Appendix S11 Potential effect modifiers examined by network meta-regression models.**

| Study characteristics | B | 95%CI | SD |
| --- | --- | --- | --- |
| Sample size | 1.52 | (-0.12, 3.16) | 0.84 |
| Country | -0.52 | (-1.84,0.84) | 0.68 |
| Publication year | -0.23 | (-1.49, 1.03) | 0.64 |
| Gender ratio | -0.05 | (-1.26, 1.17) | 0.62 |
| Mean age | 0.01 | (-1.71,1.00) | 0.69 |
| Presence of organ failure | 1.03 | (-0.29, 2.33) | 0.67 |
| Extent of necrosis≥30% | 0.21 | (-1.13,1.55) | 0.68 |

As shown in the table, all the CIs included zero, thus, all study characteristics had no effect on the final results of mortality in this study.

CI, confidence interval; SD, standard deviation

**Appendix S12 Adjusted funnels of mortality assessing for reporting bias**


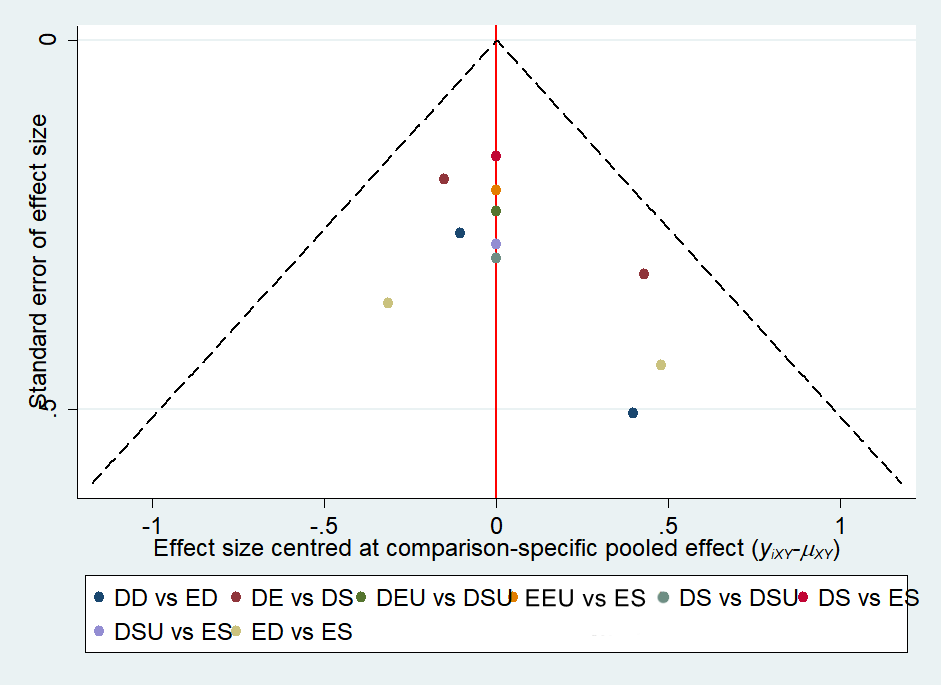


Egger’s test, p = 0.4144

CINeMA, Confidence in Network Meta-Analysis; DD, delayed drainage; DE, delayed endoscopic debridement; DEU, delayed endoscopic step-up approach; DS, delayed surgery; DSU, delayed surgical step-up approach; ED, early drainage; EEU, early endoscopic step-up approach; ES, early surgery; GRADE, Grading of Recommendations Assessment, Development, and Evaluation

**Appendix S13 GRADE ratings for the network using the CINeMA (Confidence in Network Meta-Analysis) program**

1. **Mortality**

| Comparison | Number of studies | Within study bias | Reporting bias | Indirectness | Imprecision | Heterogeneity | Incoherence | Confidence rating |
| --- | --- | --- | --- | --- | --- | --- | --- | --- |
| Mixed evidence | | | | | | | | |
| DD:ED | 2 | Some concerns | Low risk | No concerns | No concerns | No concerns | No concerns | Moderate |
| DE:DS | 1 | Some concerns | Low risk | No concerns | No concerns | No concerns | No concerns | Moderate |
| DEU:DSU | 1 | No concerns | Low risk | No concerns | No concerns | No concerns | No concerns | Moderate |
| DS:DSU | 1 | Some concerns | Some concerns | No concerns | No concerns | No concerns | No concerns | Low |
| DS:ES | 1 | Some concerns | Low risk | No concerns | No concerns | Some concerns | No concerns | Low |
| DSU:ES | 1 | No concerns | Low risk | No concerns | No concerns | No concerns | No concerns | Moderate |
| ED:ES | 2 | No concerns | Low risk | Some concerns | No concerns | No concerns | No concerns | Low |
| EEU:ES | 1 | No concerns | Low risk | Some concerns | No concerns | No concerns | No concerns | Moderate |
| Indirect evidence | | | | | | | | |
| DD:DE | 0 | No concerns | Low risk | Some concerns | Some concerns | No concerns | No concerns | Low |
| DD:DEU | 0 | No concerns | Low risk | Some concerns | Some concerns | No concerns | No concerns | Moderate |
| DD:DS | 0 | No concerns | Low risk | No concerns | Some concerns | No concerns | No concerns | Moderate |
| DD:DSU | 0 | No concerns | Low risk | No concerns | Some concerns | No concerns | No concerns | Moderate |
| DD:EEU | 0 | No concerns | Low risk | No concerns | Major concerns | Some concerns | No concerns | Low |
| DD:ES | 0 | No concerns | Low risk | No concerns | No concerns | Some concerns | No concerns | Moderate |
| DE:DEU | 0 | No concerns | Low risk | Some concerns | No concerns | Some concerns | No concerns | Low |
| DE:DSU | 0 | No concerns | Low risk | Some concerns | No concerns | No concerns | No concerns | Moderate |
| DE:ED | 0 | No concerns | Low risk | No concerns | Some concerns | No concerns | No concerns | Moderate |
| DE:EEU | 0 | No concerns | Low risk | No concerns | Some concerns | No concerns | No concerns | Moderate |
| DE:ES | 0 | No concerns | Low risk | Some concerns | No concerns | Some concerns | No concerns | Low |
| DEU:DS | 0 | No concerns | Low risk | No concerns | No concerns | Some concerns | No concerns | Moderate |
| DEU:ED | 0 | No concerns | Low risk | Some concerns | Some concerns | No concerns | No concerns | Low |
| DEU:EEU | 0 | No concerns | Low risk | No concerns | No concerns | Some concerns | No concerns | Moderate |
| DEU:ES | 0 | No concerns | Low risk | Some concerns | No concerns | Some concerns | No concerns | Low |
| DS:ED | 0 | No concerns | Low risk | Some concerns | Some concerns | No concerns | No concerns | Low |
| DS:EEU | 0 | No concerns | Low risk | Major concerns | Some concerns | No concerns | No concerns | Low |

1. **Major complications**

| Comparison | Number of studies | Within study bias | Reporting bias | Indirectness | Imprecision | Heterogeneity | Incoherence | Confidence rating |
| --- | --- | --- | --- | --- | --- | --- | --- | --- |
| Mixed evidence | | | | | | | | |
| DD:ED | 2 | Some concerns | Low risk | No concerns | No concerns | No concerns | No concerns | Moderate |
| DE:DS | 2 | Some concerns | Low risk | No concerns | No concerns | No concerns | No concerns | Moderate |
| DEU:DSU | 1 | No concerns | Low risk | No concerns | No concerns | No concerns | No concerns | Moderate |
| DEU:EEU | 1 | No concerns | Low risk | No concerns | No concerns | Some concerns | No concerns | Moderate |
| DS:DSU | 1 | Some concerns | Some concerns | No concerns | No concerns | No concerns | No concerns | Low |
| DS:ES | 1 | Some concerns | Low risk | No concerns | No concerns | Some concerns | No concerns | Low |
| DSU:ES | 1 | No concerns | Low risk | No concerns | No concerns | No concerns | No concerns | Moderate |
| ED:ES | 2 | No concerns | Low risk | Some concerns | No concerns | No concerns | No concerns | Low |
| EEU:ES | 1 | No concerns | Low risk | Some concerns | No concerns | No concerns | No concerns | Moderate |
| Indirect evidence | | | | | | | | |
| DD:DE | 0 | No concerns | Low risk | Some concerns | Some concerns | No concerns | No concerns | Low |
| DD:DEU | 0 | No concerns | Low risk | Some concerns | Some concerns | No concerns | No concerns | Moderate |
| DD:DS | 0 | No concerns | Low risk | No concerns | Some concerns | No concerns | No concerns | Moderate |
| DD:DSU | 0 | No concerns | Low risk | No concerns | Some concerns | No concerns | No concerns | Moderate |
| DD:EEU | 0 | No concerns | Low risk | No concerns | Major concerns | Some concerns | No concerns | Low |
| DD:ES | 0 | No concerns | Low risk | No concerns | No concerns | Some concerns | No concerns | Moderate |
| DE:DEU | 0 | No concerns | Low risk | Some concerns | No concerns | Some concerns | No concerns | Low |
| DE:DSU | 0 | No concerns | Low risk | Some concerns | No concerns | No concerns | No concerns | Moderate |
| DE:ED | 0 | No concerns | Low risk | No concerns | Some concerns | No concerns | No concerns | Moderate |
| DE:EEU | 0 | No concerns | Low risk | No concerns | Some concerns | No concerns | No concerns | Moderate |
| DE:ES | 0 | No concerns | Low risk | Some concerns | No concerns | Some concerns | No concerns | Low |
| DEU:DS | 0 | No concerns | Low risk | No concerns | No concerns | Some concerns | No concerns | Moderate |
| DEU:ED | 0 | No concerns | Low risk | Some concerns | Some concerns | No concerns | No concerns | Low |
| DEU:ES | 0 | No concerns | Low risk | Some concerns | No concerns | Some concerns | No concerns | Low |
| DS:ED | 0 | No concerns | Low risk | Some concerns | Some concerns | No concerns | No concerns | Low |
| DS:EEU | 0 | No concerns | Low risk | Major concerns | Some concerns | No concerns | No concerns | Low |

DD, delayed drainage; DE, delayed endoscopic debridement; DEU, delayed endoscopic step-up approach; DS, delayed surgery; DSU, delayed surgical step-up approach; ED, early drainage; EEU, early endoscopic step-up approach; ES, early surgery
